# An intersectional lens on young leaders: bias toward young women and young men in leadership positions

**DOI:** 10.3389/fpsyg.2023.1204547

**Published:** 2023-08-16

**Authors:** Christoph Daldrop, Claudia Buengeler, Astrid C. Homan

**Affiliations:** ^1^Department of Human Resource Management and Organization, Faculty of Business, Economics and Social Sciences, Kiel University, Kiel, Germany; ^2^Work and Organizational Psychology, Faculty of Social and Behavioral Sciences, University of Amsterdam, Amsterdam, Netherlands

**Keywords:** leadership, young age, gender, status, intersectionality, ageism, social dominance orientation

## Abstract

Research has recognized age biases against young leaders, yet understanding of how gender, the most frequently studied demographic leader characteristic, influences this bias remains limited. In this study, we examine the gender-specific age bias toward young female and young male leaders through an intersectional lens. By integrating intersectionality theory with insights on status beliefs associated with age and gender, we test whether young female and male leaders face an interactive rather than an additive form of bias. We conducted two preregistered experimental studies (*N*_1_ = 918 and *N*_2_ = 985), where participants evaluated leaders based on age, gender, or a combination of both. Our analysis reveals a negative age bias in leader status ascriptions toward young leaders compared to middle-aged and older leaders. This bias persists when gender information is added, as demonstrated in both intersectional categories of young female and young male leaders. This bias pattern does not extend to middle-aged or older female and male leaders, thereby supporting the age bias against young leaders specifically. Interestingly, we also examined whether social dominance orientation strengthens the bias against young (male) leaders, but our results (reported in the SOM) are not as hypothesized. In sum, our results emphasize the importance of young age as a crucial demographic characteristic in leadership perceptions that can even overshadow the role of gender.

## 1. Introduction

As the workforce diversifies in age and young-led tech industries continue to expand their influence on the economy, an increasing number of skilled young professionals are stepping into leadership positions. In fact, 38% of American workers now report to a young leader (Kaufman, 2017). Consequently, examining how young leaders are perceived has become increasingly important for organizational scholars.

Research has identified negative perception biases against young adults in leadership positions (e.g., Buengeler et al., 2016; Kunze and Menges, 2016). This is consistent with studies on gender and leadership, which reveal similar biases against female leaders (Ridgeway, 2001; Eagly and Karau, 2002; Rudman et al., 2012). These biases can be explained, in part, by the lower status beliefs associated with demographic characteristics such as young age or gender (i.e., diffuse status characteristics, e.g., Lianidou and Zheng, 2022). Status beliefs can be defined as “widely held cultural beliefs that associate greater social significance and general competence […] with one category of a social distinction over another” (Ridgeway, 2001, p. 638). However, our understanding of the status beliefs associated with the intersections of demographic characteristics, such as age *and* gender, is still limited.

To thoroughly examine the age bias toward young leaders, it is crucial to adopt an *intersectional lens*, especially considering the well-documented bias women face in leadership positions (e.g., Koenig et al., 2011). Intersectionality offers a framework for investigating how multiple aspects of an individual’s identity, such as age and gender, intersect and influence their experiences and challenges (Crenshaw, 1990; Cole, 2009). However, the nature of intersectional bias toward young women and men in leadership is not understood yet. When people apply an intersectional lens, do young female leaders face bias due to their young age *and* female gender added together, resulting in a “double jeopardy” effect (additive effect; e.g., Berdahl and Moore, 2006; Nelson, 2016)? Or do they face bias based on only one (or neither) of these factors, resulting in an “intersectional escape” (interactive effect; e.g., Purdie-Vaughns and Eibach, 2008; Martin et al., 2019)? Similarly, does the gender of young male leaders counterbalance a potential age bias (i.e., additive effect), or is bias still present as it may primarily be due to their young age (i.e., interactive effect)?

In this study, we take an intersectional approach to examine age bias in people’s perceptions of leader status—including respect, prominence, and prestige (e.g., Djurdjevic et al., 2017). To do so, we develop theory on biases against young female and male leaders integrating status characteristics reasoning (status characteristics theory; Berger et al., 1977; Ridgeway et al., 1985) with intersectionality theory and research (Hall et al., 2019; Petsko et al., 2022). To predict the intersectional bias, we further theorize on group prototypicality concerning age and gender for young women and men. Group prototypicality helps to explain why certain group members are considered more representative of a specific group than others (Purdie-Vaughns and Eibach, 2008; Hall et al., 2019). Comparing people’s leader status perceptions as seen through different lenses—age (i.e., young leaders), gender (i.e., female and male leaders), and age *and* gender (i.e., young female and male leaders)—allows us to determine the nature of the intersectional bias toward young female and male leaders.

With our research, we offer two important contributions to research on age bias in leadership. First, we extend the growing literature on intersectionality in leadership research (e.g., Rosette et al., 2016, 2018) to age and gender, exploring how these factors may influence the perceptions of leaders. By comparing the intersection of age with gender to both single group categories, we can determine which aspect carries greater influence in evaluations of young female and young male leaders. Second, we deepen understanding of age bias toward young adults in leader roles from a status characteristics perspective and reveal lower leader status as a critical correlate. Through empirical evidence from two pre-registered experimental studies (*N*_1_ = 918 and *N*_2_ = 985), we reveal the relative importance of young age as a key demographic characteristic, surpassing gender, the most studied demographic leader characteristic (Lianidou and Zheng, 2022).

## 2. An intersectional lens on young leaders

In psychological and management science*, intersectionality* (Crenshaw, 1990; Cole, 2009) refers to how biases and stereotypes manifest simultaneously across multiple group categories, such as race, gender, or age (e.g., Rosette et al., 2018). When group categories, like age or gender, intersect, they can form a new category with unique biases and stereotypes that may be separate from the original group categories.

The lens-based perspective of intersectional stereotyping (Petsko et al., 2022) suggests that people use separate lenses, such as gender, age, or intersectionality, to perceive and categorize others. Importantly, according to this model, only one lens is used at a time during perception and stereotyping. The choice of lens depends on factors like *accessibility* (how easily it can be retrieved from memory), *fit* (how well it aligns with the specific context), *distinctiveness* (how noticeable the group category is), and the individual’s *goal* (their motivation to categorize based on one group category over another; Petsko and Bodenhausen, 2020; Petsko et al., 2022).

In general, people do not use an intersectional, age, or gender lens by default (Petsko et al., 2022). Instead, they may opt for the most accessible, salient, and contextually fitting lens. When evaluating young female and young male leaders, individuals may apply lenses based on age, gender, or their intersection. People may perceive young female leaders through a gender lens, categorizing them as *women*, or through an age lens, categorizing them as *young individuals*. Alternatively, people may apply an intersectional lens, categorizing young female leaders as *young women*.

The lens people adopt to perceive leaders may have consequences, as each lens emphasizes specific attributes and *status ascriptions* tied to a particular group (Petsko et al., 2022). Unlike specific status, which arises from well-defined attributes directly related to ability, education, or functional background, *ascribed* status is based on diffuse characteristics like gender or age (e.g., Lianidou and Zheng, 2022). Indeed, status characteristics theory posits that people ascribe higher status to certain social groups (e.g., men, older adults) and lower status to others (e.g., women, young adults; Berger et al., 1977; Ridgeway et al., 1985). As ascribed status is independent of an individual’s skills and expertise, it can lead to *biased* expectations and decisions regarding leaders.

Both gender and age bias involve one group being associated with greater social significance, competence, and status than others. Men, who tend to hold dominant positions in society, are typically seen as the gender-neutral standard, whereas women are viewed as more gender-specific (Bailey et al., 2019). Dominant positions afford men more access to power and resources than women, leading to greater respect and prestige (Ridgeway, 1991). Regarding age, research indicates that older adults usually possess more social power and status than young adults (e.g., Sidanius and Pratto, 1999; Triana et al., 2017). Consequently, there is an unequal distribution of social status among gender and age groups, with men and older adults generally having higher status than women and young adults.

The primary difference between gender and age bias lies in the roots of their respective status characteristics (Martin and North, 2022). Gender is perceived as a more static status characteristic, with the categories of men, women (and nonbinary individuals).^1^ In contrast to gender, age is a more dynamic and continuous status characteristic. Age-based status ascriptions—both positive and negative—are relevant for all individuals over time, assuming they experience a sufficient lifespan. Due to age’s dynamic nature and the natural aging process, age bias is often less acknowledged or more accepted than gender bias (Nelson, 2016; Martin and North, 2022). Individuals may believe that young adults aspiring to or holding leadership positions should “wait their turn,” and perceive it as fair that young adults do not hold leadership positions. We therefore argue that young age is even more problematic than gender regarding biases in leader status perceptions. In the following sections, we will theorize on the intersection of age and gender in relation to leader evaluations based on their respective group prototypicality.

### 2.1. Bias toward young female leaders

Group prototypicality, referring to the extent to which an individual is perceived as a typical or representative member of a specific social group (e.g., Rosch and Mervis, 1975; Rosch, 1978), plays a crucial role in leader evaluations. More visible, and easily categorized, prototypical members can face an amplified bias, whereas less prototypical members may experience a diluted bias (Hall et al., 2019). Leaders’ prototypicality in terms of age and gender may thus shape an intersectional bias toward young female leaders.

We suggest that within their gender group, young women are perceived as more prototypical of the category “women” than older women. Stereotypes associated with women (e.g., femininity) may align more closely with stereotypes associated with young age (e.g., attractiveness, vitality) than older age (e.g., decreased attractiveness; Kite et al., 2005). This greater congruence between stereotypes of women and those of a young age makes young women more salient and representative of their gender group compared to older women (Hall et al., 2019). Consequently, young women may be perceived as prototypical for the category of women, while older women are seen as less prototypical, thereby diluting gender-based status ascriptions for older women (i.e., intersectional escape; Martin et al., 2019).

However, we assume that within the young age group, women are perceived as less prototypical of the category “young adults” compared to men. Specifically, the gender-based social hierarchy (Ridgeway, 1991) may cause people to view young women as less representative members of the younger age group compared to young men. This perception arises because men, due to their dominant societal position, form the gender-neutral standard, while women are seen as more gender-specific (e.g., Bailey et al., 2019). Moreover, stereotypes associated with younger age (e.g., self-confident, assertive; Kite et al., 2005) may align more closely with stereotypes linked to men (i.e., agentic, assertive) than with those connected to women (i.e., communal attributes, e.g., Eagly et al., 2020). As a result, young men are highly prototypical for the young age group, amplifying age status ascriptions. In contrast, young women’s less prototypical status may dilute age-based status ascriptions.

Building on young women’s gender prototypicality, we anticipate that young female leaders will encounter a non-additive, interactive form of bias. We propose that young female leaders face relatively more gender-based than age-based status ascriptions. Although both gender and age are likely to contribute negatively to leader status ascriptions, we posit that young age outweighs gender in terms of its impact on status ascriptions. This is due to the dynamic and continuous nature of age as a status characteristic, compared to the more static nature of gender as a status characteristic (e.g., Martin and North, 2022). In other words, for women leaders we propose that it is more detrimental to be perceived as young rather than as a young female (i.e., due to diluted status ascriptions for young female leaders), whereas being seen as a female leader should yield less negative perceptions than being perceived as a young leader (as being young is more problematic than being female). Consequently, we hypothesize that “young leaders” (i.e., being viewed through an age lens only) receive more negative status ascriptions than “young female leaders” (i.e., being viewed through an intersectional lens; *H1a*). Additionally, the categorization as “young female leaders” might be assessed more negatively than “female leaders” (i.e., being viewed through a gender lens only; *H1b*) due to (diluted) age-based status ascriptions for young female leaders. We hypothesize:

*H1a/b: Young female leaders are assigned (a) higher leadership status than young leaders in general but (b) lower leadership status than female leaders in general.* ^2^

### 2.2. Bias toward young male leaders

So far, our theorizing has centered on the intersectional bias toward young female leaders. However, we argue that there may also be bias against young male leaders, manifested in diminished perceptions of their leader status. We propose that this bias could emerge not only when people evaluate young male leaders through an age lens (i.e., as young adults) but also when applying an intersectional lens (i.e., as young men).

We suggest that within their gender group, young men are perceived as more prototypical of the category “men” than older men. Stereotypes associated with men (i.e., agentic, assertive, e.g., Eagly et al., 2020) more closely align with stereotypes associated with younger individuals (e.g., self-confident, assertive) than those related to older individuals (i.e., being less agentic, e.g., Kite et al., 2005). Consequently, the gender prototypicality of young men stems from a greater overlap of stereotypes associated with men and young adults. There is less overlap between stereotypes associated with men and older adults, leading to lower gender prototypicality for older men (Hall et al., 2019).

Compared to young women, the “male as the standard paradigm” and the gender-based social hierarchy (Ridgeway, 2001; Bailey et al., 2019) further suggest that young men are seen as prototypical members of the young age group.

Based on our reasoning regarding the age and gender prototypicality of young men, we expect bias against young male leaders. Both age and gender should contribute to the intersectional bias against young male leaders, but in different ways. While being a man is generally associated with higher status (e.g., Ridgeway, 2001), being young is typically linked to lower status (e.g., Triana et al., 2017). Therefore, only young age should negatively impact the status perception of young male leaders. As such, we hypothesize that “young male leaders” (i.e., being viewed through an intersectional lens) receive more negative evaluations than “male leaders” (i.e., being viewed through a gender lens only), due to lower age-based status ascriptions. Since young male leaders are seen as highly prototypical for the young age group, we do not expect a difference in leader status ascriptions between young male leaders and young leaders in general. We hypothesize:

*H2: Young male leaders are assigned lower leadership status than male leaders in general.*

## 3. Overview of the research

To test our hypotheses, we conducted two experimental studies (*N*_1_ = 918 and *N*_2_ = 985). Both studies adhered to the American Psychological Association (APA) guidelines and obtained approval from the University of Amsterdam’s Economics & Business Ethics Committee (protocol numbers: EC 20220209020230 [Study 1], EB-1013 [Study 2]). We determined appropriate sample sizes *a priori* and performed no statistical analyses until all data were collected.

We pre-registered Study 1 using the Psychological Research Preregistration-Quantitative Template (PRP-QUANT; Bosnjak et al., 2022) on the PsychArchives repository.^3^ For Study 2, we used the AsPredicted template and pre-registered the study via AsPredicted.^4^ We noted the cases where participants were dropped from the sample in line with our pre-registered exclusion criteria.

Additional analyses and results related to a pre-registered hypothesis about the influence of social dominance orientation are provided in the Supplementary material file (sections #2 and #4). The results did not support our hypothesis about the strengthening effect of social dominance orientation in the comparison of young men and men regarding leader status. An interaction effect emerged, however, between social dominance orientation and the comparison of young men and young women’s perceived leader status. This finding provides suggestive support for the *subordinate male target hypothesis* (i.e., people with a preference for group-based hierarchy perceive especially male members of non-dominant groups as a threat to their dominant position; e.g., Sidanius and Pratto, 1999). Interestingly, we found suggestive evidence that individuals with a higher social dominance orientation exhibit a stronger bias against young adults. This bias is evident in the lower status ascribed to young leaders compared to male leaders (Study 1, see Supplementary material section #2.2.3), and to young leaders compared to middle-aged leaders (Study 2, see Supplementary material section #4.1.2). The pre-registration documents and Supplementary material can be accessed via the Open Science Framework (OSF) platform using the following link: https://osf.io/gmqt9/?view_only=81b8ac4b5f684d34a311a1c663bfad11.

## 4. Study 1

In Study 1, we examine our hypotheses regarding the intersection of young age and gender. Specifically, we assess the presence of a gender-specific age bias toward young female leaders (*H1a/b*) and young male leaders (*H2*) by comparing the intersectional lenses to gender and age lenses.

### 4.1. Methods

#### 4.1.1. Participants

Participants were recruited via the ZPID’s PsychLab^5^ in collaboration with panel provider Respondi.^6^ Data was collected from a heterogeneous sample of U.S. citizens aged 25–69 to ensure generalizability across workplace age groups.^7^ For adequate representation of evaluators from various ages and genders within the overall sample, we divided our sample into six evaluator subgroups. Cross-quotas were employed for evaluator age groups and gender across target conditions (i.e., young women and men: 25–39 years; middle-aged women and men: 40–54 years; older women and men: 55–69 years).^8^

An *a priori* power analysis, based on the average effect size from similar previous studies (*f^2^* = 0.04), indicated that 465 participants were needed to achieve 90% power to detect the anticipated small effect at *α* = 0.05 (Faul et al., 2009). In our analyses, we pre-registered comparisons between one target group (e.g., young female targets) and two control groups (e.g., young targets, female targets). We aimed to recruit 180 participants for each target group to ensure sufficient statistical power. With five target groups, the minimum required number of participants was 900. Deviations from this goal were due to the software employed by the panel provider and outside of our control.

We collected data from 982 participants. Those with incomplete data (i.e., participants who dropped out) were excluded. To ensure high data quality, we excluded 64 participants who incorrectly answered at least one of the two pre-registered understanding and diligence checks (e.g., Gloor et al., 2020; Arthur et al., 2021). Specifically, we excluded 15 participants with insufficient English language proficiency (i.e., those who indicated only basic communication skills/working knowledge [A1 to A2] on an English proficiency item with six response options) and 49 participants who self-reported careless responses (“*should we use your data for our scientific analyses?*,” e.g., Aust et al., 2013).

Our analyses were conducted using a final sample size of 918 participants. All participants received the same predetermined payment based on the expected average completion time. The sample was balanced according to our quotas for gender (455 women, 463 men) and age groups (296 young, 311 middle-aged, 311 older). Participants had an average age of 48.20 years (*SD* = 12.70) and were predominantly White (725 White, 59 Asian, 52 Black, 50 Latin, 9 Native American, and 23 unspecified). Most participants were employed (627 participants) in various occupations such as education, manufacturing, and retail, working an average of 37.5 h per week (*SD* = 10.8). Three hundred and four participants had supervisory responsibilities currently (256 participants) or in former positions (48 participants). Participants reported their political orientation using the proxy of right-wing ideology (six-item right-wing authoritarianism scale; Aichholzer and Zeglovits, 2015). In our sample, 30.2% of participants favored right-wing ideology (23.0% slightly, 7.2% somewhat/strongly agree), 50.2% were neutral, and 19.6% opposed right-wing ideologies (14.3% slightly, 5.3% somewhat or strongly disagreed). This distribution corresponds with the political makeup of the general U.S. population (Hawkins et al., 2019).

#### 4.1.2. Design and procedure

We conducted an experimental study using a between-subjects design to evaluate ratings through different lenses: gender (women, men), age (young adults), and intersectionality (young women, young men). The inclusion of gender and age lenses (women, men, young adults) was crucial to determine whether there is an interactive bias resulting from a combination of age and gender biases in the intersectional categories of young women and young men. Participants were randomly assigned to one of the five target groups: women, men, young individuals, young women, and young men.

First, participants read a brief scenario description before rating their assigned target: “On the following pages, you will find a series of attributes commonly used to characterize people in general. We would like you to use this list to tell us how DESIRABLE it is in the workplace for [condition-dependent target group member] to possess the following characteristics. That is, regardless of how [target group] actually is, we want to know how people in the workplace think [target group] SHOULD be. In making your judgments, it may be helpful to imagine that you are about to meet a person in the workplace for the first time, and the only thing you know in advance is that the person is a [target group].” The scenario description was adapted from Schein (1973, 1975) and has been successfully employed in previous research targeting various groups (e.g., Ryan et al., 2011; Morgenroth et al., 2021). For young women, young men, and young adults, the displayed age range was set between 25 and 39 years, following our definition of young adults in the workplace. After rating the attributes (for which results are reported in section #2.3.4 of the Supplementary material as they were not core to our research question), participants were instructed to imagine that the [target group] was their current leader. They rated their target group on our pre-registered dependent variable, perceived leader status. Additionally, we measured other variables not central to our pre-registered hypotheses, such as perceived leader effectiveness and leader liking (see section #2.3.2 in the Supplementary material). The study concluded with a demographic questionnaire.

#### 4.1.3. Measures

##### 4.1.3.1. Perceived leader status

We measured perceived leader status using four items from a scale by Djurdjevic et al. (2017). Participants responded to statements such as, “*This leader possesses high status in my organization*,” “*This leader occupies a respected position in my organization*,” “*This leader has a position of prestige in my organization*,” and “*This leader possesses a high level of prominence in my organization*.” Participants used a 7-point scale to indicate perceived leader status (1 = strongly disagree to 7 = strongly agree). Cronbach’s alpha for the four leader status items was 0.93.

##### 4.1.3.2. Control variables

We incorporated the evaluator’s age and gender as control variables. Research has demonstrated that older evaluators exhibit stronger prescriptive stereotypes for young and older targets (e.g., De Paula Couto and Rothermund, 2019). As individuals age, they may better understand societal expectations for themselves and others, leading to stronger prescriptive stereotypes toward young individuals due to increased experience with and exposure to social norms (Kornadt et al., 2017). Evaluator age was measured as a continuous variable and mean-centered for our analyses. Regarding gender, research indicated that men tend to hold stronger prescriptive stereotypes than women (Martin et al., 2019). Participants identified their own gender using a single item (“please indicate your gender”; woman, man).

#### 4.1.4. Manipulation checks

We asked participants to indicate the age they were thinking about when evaluating the leader (“*In the previous questions, we asked you to think about a specific person as your leader. What age do you think this person would typically have?*”). The differences between the conditions for which age information was presented (i.e., young) versus for those conditions for which no age information was presented were significant. The indicated gender of the leader did not influence the age ascribed to the leader by the participants (see Table 1).

**Table 1:** Study 1: Means and standard deviations of typical age rating for the specific target groups.

	No age info	Young
No gender info	–	30.43^b^ (3.77)
Women	40.61^a^ (7.40)	30.08^b^ (3.69)
Men	41.73^a^ (7.53)	30.23^b^ (4.14)

*N* = 918. Means that share superscripts did not differ at *p* < 0.05 in independent sample *t*-tests. Standard deviations are reported in brackets.

### 4.2. Results

#### 4.2.1. Descriptive statistics

We report correlations among the study variables, including demographics (evaluator age, evaluator gender), independent variables (dummy variables for the target groups), and leader evaluations (perceived leader status) in Table 2. Further, we provide mean ratings of perceived leader status by target condition in Table 3.

**Table 2:** Study 1: Correlations between study variables.

	1	2	3	4	5	6	7
1. Evaluator age	–						
2. Evaluator gender^a^	0.01	–					
3. Women^b^	−0.01	0.00	–				
4. Men^b^	−0.02	0.01	−0.28**	–			
5. Young adults^b^	0.06	0.04	−0.26**	−0.26**	–		
6. Young women^b^	0.00	−0.01	−0.25**	−0.25**	−0.24**	–	
7. Young men^b^	−0.03	−0.05	−0.25**	−0.25**	−0.24**	−0.23**	–
8. Perceived leader status	−0.03	0.02	0.15**	0.01	−0.12**	0.03	−0.07*

*N* = 918. ^a^ Women are coded 0, men are coded 1. ^b^ Each dummy variable groups one target condition (e.g., young adults coded 1) against the other four target conditions (e.g., women, men, young women, and young men coded 0) and therefore provides only limited information regarding bivariate correlations. *p < 0.05; **p < 0.01.

**Table 3:** Study 1: Means and standard deviations of perceived leader status for the specific target groups.

	No age info	Young
No gender info	–	5.17 (1.16)
Women	5.79 (1.16)	5.53 (1.16)
Men	5.48 (1.15)	5.28 (1.11)

*N* = 918. Standard deviations are reported in brackets.

#### 4.2.2. Preliminary analyses: gender and perceived leader status

**Figure 1:**
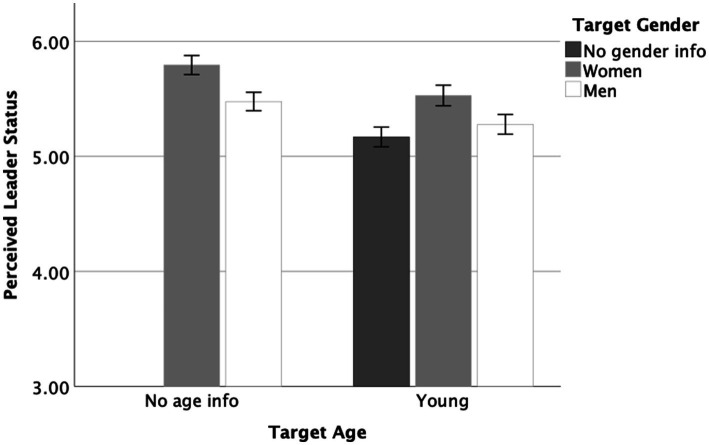
Study 1: Results for perceived leader status by target groups. Error bars represent standard errors.

Before testing our hypotheses regarding the intersectional effects of young age and gender, we conducted analyses to identify gender differences in perceived leader status for women and men (i.e., when no age information was presented). We conducted independent samples *t*-test using IBM SPSS 29. There was a difference in perceived leader status between men and women, *t*(396) = −2.74, *p* = 0.006, *d* = 0.28, with women scoring higher than men (*M_diff_* = −0.32, 95% CI [−0.54, −0.09]). We present perceived leader status ratings for different target groups in Figure 1.

#### 4.2.3. Main analyses

We tested our hypotheses using independent samples *t*-tests.^9^ In Hypotheses *H1a/b* and *H2*, perceived leader status was the dependent variable. Young women were perceived as having more leader status than young individuals, *t*(349) = 2.92, *p* = 0.004, *d* = 0.31, *M_diff_* = 0.36, 95% CI [0.12, 0.60], supporting Hypothesis *H1a*. Further, young women were perceived as having less leader status than women in general, *t*(363) = −2.17, *p* = 0.031, *d* = −0.23, *M_diff_* = −0.26, 95% CI [−0.50, −0.02], supporting Hypothesis *H1b*.

The perceived leader status did not differ between young men and men, *t*(370) = −1.68, *p* = 0.093, *d* = −0.18, *M_diff_* = −0.19, 95% CI [−0.43, 0.03], failing to support Hypothesis *H2*. Further, young men and young individuals did not differ in perceived leader status, *t*(348) = 0.90, *p* = 0.184, *d* = 0.10, *M*_*dif*f_ = 0.11, 95% CI [−0.13, 0.35].

In addition to our main analyses, we compared the intersectional categories of young women and young men to discern differences *within* this age group. Young women were perceived as having higher leader status than young men, *t*(337) = 2.03, *p* = 0.043, *d* = 0.22, *M_diff_* = 0.25, 95% CI [0.01, 0.49].

### 4.3. Discussion (Study 1)

Study 1 provides valuable insights into how different groups are perceived as leaders. Our results indicate that young female leaders are perceived as having a higher status than young leaders, which supports Hypothesis *H1a*. Further, young female leaders were seen as lower in status than female leaders, supporting Hypothesis *H1b*. However, our Hypothesis 2 was not supported. Young male leaders were not perceived as having lower status than male leaders. Finally, we found no difference between the perceived leader status of young male leaders and young leaders, in line with our expectations. Interestingly, our results also revealed a gender difference in perceived leader status, with (young) female leaders scoring higher than (young) male leaders.

To better understand whether the gender-specific age bias affects only young leaders or also applies to middle-aged and older leaders, it is crucial to compare evaluations across all three age groups. These comparisons also allow ruling out alternative explanations, such as the notion that the presence of age information, regardless of whether the leader is young, middle-aged, or older, leads to more negative leader evaluations. Without these comparisons, the assertion that young leaders are evaluated more negatively lacks an appropriate comparative framework. To determine whether the established bias is about young age or age in general and further corroborate our conclusion, we theorize on and examine the age bias toward young leaders compared to middle-aged and older leaders.

In many societies, age-based social hierarchies result in differential access to rewards, power, and privileges for people of various ages (e.g., Sidanius and Pratto, 1999; Triana et al., 2017). Older adults are often seen as having higher social status than young adults. Older, higher-status individuals are perceived as more competent due to the greater expectations regarding their contributions to a specific group. For instance, leaders’ older age can enhance their ability to influence others effectively (e.g., Buengeler et al., 2016), leading to attributions of higher status and competence.

For young leaders, this implies that people may form biased assumptions based on an individual’s young age rather than considering young individuals’ actual competence, expertise, or other factors relevant to leadership performance (e.g., education, functional background, i.e., specific status characteristics; Lianidou and Zheng, 2022). The lower-status beliefs associated with young age may entail expectations of reduced competence, resulting in limited opportunities and biased evaluations (e.g., Triana et al., 2017). Consequently, these beliefs about young individuals may diminish perceptions of respect and prestige toward young leaders in organizational settings (i.e., ascriptions of leadership status) and negatively affect their perceived competence and expertise (i.e., perceived effectiveness). Therefore, we hypothesize:

*H3a/b: Young leaders are assigned lower leadership status than (a) middle-aged leaders and (b) older leaders.*

We further investigate how the intersections of age with gender influence perceptions of leader status. While our first study focused on the intersections of young age with gender, as outlined in our Hypotheses *H1a/b* and *H2*, we also consider the alternative explanation that negative perceptions of leader status may be linked to the general presence of age information, regardless of the leader’s age. Therefore, we aim to explore the intersectional lens of middle-aged and older age with female (*RQ1a/b*) and male gender (*RQ2a/b*) by proposing the following research questions:

*RQ1a/b: Do people assign lower leadership status to (a) middle-aged female leaders and (b) older female leaders compared to female leaders in general?*

*RQ2a/b: Do people assign lower leadership status to (a) middle-aged male leaders and (b) older male leaders compared to male leaders in general?*

## 5. Study 2

In Study 2, we investigate the potential age bias against young leaders compared to middle-aged and older leaders (*H3a/b*). Second, we test our hypotheses concerning the intersection of young age and gender (*H1a/b*, *H2*) to establish generalizability. Third, we explore the intersection of middle-aged and older age with gender in an exploratory manner (*RQ1a/b* and *RQ2a/b*).

### 5.1. Methods

#### 5.1.1. Participants

An *a priori* power analysis based on the effect size from Study 1 indicated that 968 participants would be required to achieve 95% power for detecting the anticipated small to medium effect (Cohen’s *f* = 0.16) at *α* = 0.05 (Faul et al., 2009). We thus recruited 1000 participants through the panel provider CloudResearch Connect.^10^ Participants with incomplete data (i.e., those who dropped out) were excluded from the study.

To maintain high data quality, we excluded 15 participants who failed to correctly answer at least one of the two pre-registered understanding and diligence checks (e.g., Gloor et al., 2020; Arthur et al., 2021). This group consisted of five participants with inadequate English language proficiency and 10 participants who self-reported careless responses both determined as in Study 1.

Whereas in Study 1, all age groups were represented equally, in Study 2, we aimed to obtain a representative sample of the U.S. by implementing quotas based on recent U.S. census data, which considered factors such as gender, age, race, and political orientation (U.S. Census Bureau, 2021). Any deviations from the target demographics may have resulted from the panel provider’s software limitations and were beyond our control. Our sample included 499 women, 483 men, and three individuals who identified as neither male nor female. The participants’ mean age was 42.35 years (*SD* = 13.82), ranging from 18 to 69 years. Regarding racial background, 74.9% identified as White, 11.2% as Black, and 13.1% as Asian, Native American, or another race. Political orientation was distributed as 39.3% conservative, 29.1% moderate, and 31.6% liberal. This methodology ensured generalizable findings that accurately represent the diverse U.S. population.

#### 5.1.2. Design and procedure

We conducted an experimental study with a between-subjects design where participants evaluated a leader belonging to a specific target group. The study is organized into three sub-studies, each focusing on different aspects of the target: (a) gender (two conditions: women, men), (b) age (three conditions: young adult, middle-aged adult, older adult), and (c) intersections of age and gender (six conditions: young women, young men, middle-aged women, middle-aged men, older women, older men).

Participants read the following scenario before rating their target group regarding leader evaluation measures: “On the following pages, you will find a series of statements and questions. When answering these questions, please imagine working in an organization where a [condition-dependent target group member] is your current leader. In making your judgments, it may be helpful to imagine that you are about to meet your leader for the first time, and the only thing you know in advance is that your leader is a [target group]. How do you feel about a [target group] as your current leader?”

#### 5.1.3. Measures

We used the same leader evaluation measures as those employed in Study 1. These measures include perceived leader status (Djurdjevic et al., 2017), perceived leader effectiveness (Giessner and van Knippenberg, 2008; Gündemir et al., 2019), and leader liking (Rudman et al., 2012). Cronbach’s alpha for perceived leader status was 0.95. In section #3.1 of the Supplementary material, we provide details on perceived leader effectiveness and leader liking.

#### 5.1.4. Manipulation checks

We asked participants to specify the age they had in mind in their leader evaluations (The question was: “*In the previous questions, we asked you to think about a specific person as your leader. What age do you think this person would typically have?*”). Like in Study 1, we only found significant differences in specified age between the different age conditions. There was no difference in age ratings for male and female leaders (see Table 4). Furthermore, age ratings did not vary within the respective age groups (e.g., young, middle-aged, older), independent of whether these target groups were presented with or without gender information.

**Table 4:** Study 2: Means and standard deviations of typical age rating for the specific target groups.

	No age info	Young	Middle-aged	Older
No gender info	–	27.54^b^ (5.47)	45.90^c^ (5.61)	56.65^d^ (8.43)
Women	40.98^a^ (7.40)	27.80^b^ (4.33)	45.61^c^ (6.06)	54.90^d^ (6.65)
Men	41.62^a^ (6.97)	28.77^b^ (8.84)	45.28^c^ (5.67)	56.37^d^ (7.54)

*N* = 985. Means that share superscripts did not differ at *p* < 0.05 in independent sample *t*-tests. Standard deviations are reported in brackets.

### 5.2. Results

#### 5.2.1. Descriptive statistics

We report correlations among the study variables, including demographics (evaluator age, evaluator gender), independent variables (dummy variables for the target groups), and leader evaluations (perceived leader status) in Table 5. We provide mean ratings of the leader evaluations by target condition in Table 6.

**Table 5:** Study 2: Correlations between study variables.

	1	2	3	4	5	6	7	8	9	10	11	12	13
1. Evaluator age	–												
2. Evaluator gender^a^	−0.12**	–											
3. Women^b^	−0.01	−0.03	–										
4. Men^b^	−0.01	−0.01	−0.10**	–									
5. Young adults^b^	−0.01	0.02	−0.10**	−0.10**	–								
6. Middle-aged adults^b^	0.01	0.02	−0.10**	−0.10**	−0.10**	–							
7. Older adults^b^	−0.04	0.05	−0.10**	−0.10**	−0.10**	−0.10**	–						
8. Young women^b^	0.00	−0.04	−0.10**	−0.10**	−0.10**	−0.10**	−0.10**	–					
9. Young men^b^	0.00	−0.01	−0.10**	−0.10**	−0.10**	−0.10**	−0.10**	−0.10**	–				
10. Middle-aged women^b^	0.02	−0.03	−0.10**	−0.10**	−0.10**	−0.10**	−0.10**	−0.10**	−0.10**	–			
11. Middle-aged men^b^	0.05	−0.04	−0.10**	−0.10**	−0.10**	−0.10**	−0.10**	−0.10**	−0.10**	−0.10**	–		
12. Older women^b^	−0.01	0.09**	−0.10**	−0.10**	−0.10**	−0.10**	−0.10**	−0.10**	−0.10**	−0.10**	−0.10**	–	
13. Older men^b^	−0.01	−0.01	−0.10**	−0.10**	−0.10**	−0.10**	−0.10**	−0.10**	−0.10**	−0.10**	−0.10**	−0.10**	–
14. Perceived leader status	−0.05	0.01	0.07*	0.01	−0.10**	0.03	0.09**	−0.08*	−0.17**	−0.01	0.00	0.06	0.09**

*N* = 985. ^a^ Women are coded 0, men are coded 1. ^b^ Each dummy variable groups one target condition (e.g., young adults coded 1) against the other ten target conditions (i.e., all coded 0) and therefore provides only limited information regarding bivariate correlations. *p < 0.05; **p < 0.01.

**Table 6:** Study 2: Means and standard deviations of perceived leader status for the specific target groups.

	No age info	Young	Middle-aged	Older
No gender info	–	5.06 (1.20)	5.51 (0.98)	5.74 (0.85)
Women	5.66 (1.03)	5.13 (1.32)	5.38 (1.23)	5.62 (1.15)
Men	5.43 (0.94)	4.81 (1.17)	5.42 (0.94)	5.70 (0.85)

*N* = 985. Standard deviations are reported in brackets.

#### 5.2.2. Preliminary analyses: gender and perceived leader status

Before testing our hypotheses, we again conducted preliminary analyses to identify gender differences in leader evaluations for women and men (i.e., when no age information was presented). We conducted independent samples *t*-tests using IBM SPSS 29. The significantly higher leader status of women as compared to men in Study 1 did not replicate, even though the direction of findings was the same, *t*(179) = 1.57, *p* = 0.12, *d* = 0.23, *Md_iff_* = 0.23, 95% CI [−0.06, 0.52].

#### 5.2.3. Main analyses

##### 5.2.3.1. Age and perceived leader status

In support of *H3a* and *H3b*, perceived leader status differed between young adults and middle-aged adults, *t*(179) = −2.79, *p* = 0.003, *d* = 0.42, *M_diff_* = −0.45, 95% CI [−0.77, −0.13], and between young adults and older adults, *t*(177) = −4.36, *p* < 0.001, *d* = 0.65, *M_diff_* = −0.45, 95% CI [−0.99, −0.37]. Middle-aged and older adults did not differ in perceived leader status, *t*(176) = −1.64, *p* = 0.103, *d* = −0.25, *M_diff_* = −0.23, 95% CI [−0.50, 0.05].

##### 5.2.3.2. Intersections of young age with gender

An independent sample *t*-test indicated no differences in perceived leader status between young women and young individuals, *t*(178) = 0.40, *p* = 0.692, *d* = 0.06, *M_diff_* = 0.07, 95% CI [−0.30, 0.44], which does not support Hypothesis *H1a*. Young women were seen as having lower leader status than women in general, *t*(179) = −2.98, *p* = 0.003, *d* = −0.44, *M_diff_* = −0.52, 95% CI [−0.87, −0.18], supporting Hypothesis *H1b.*

**Figure 2:**
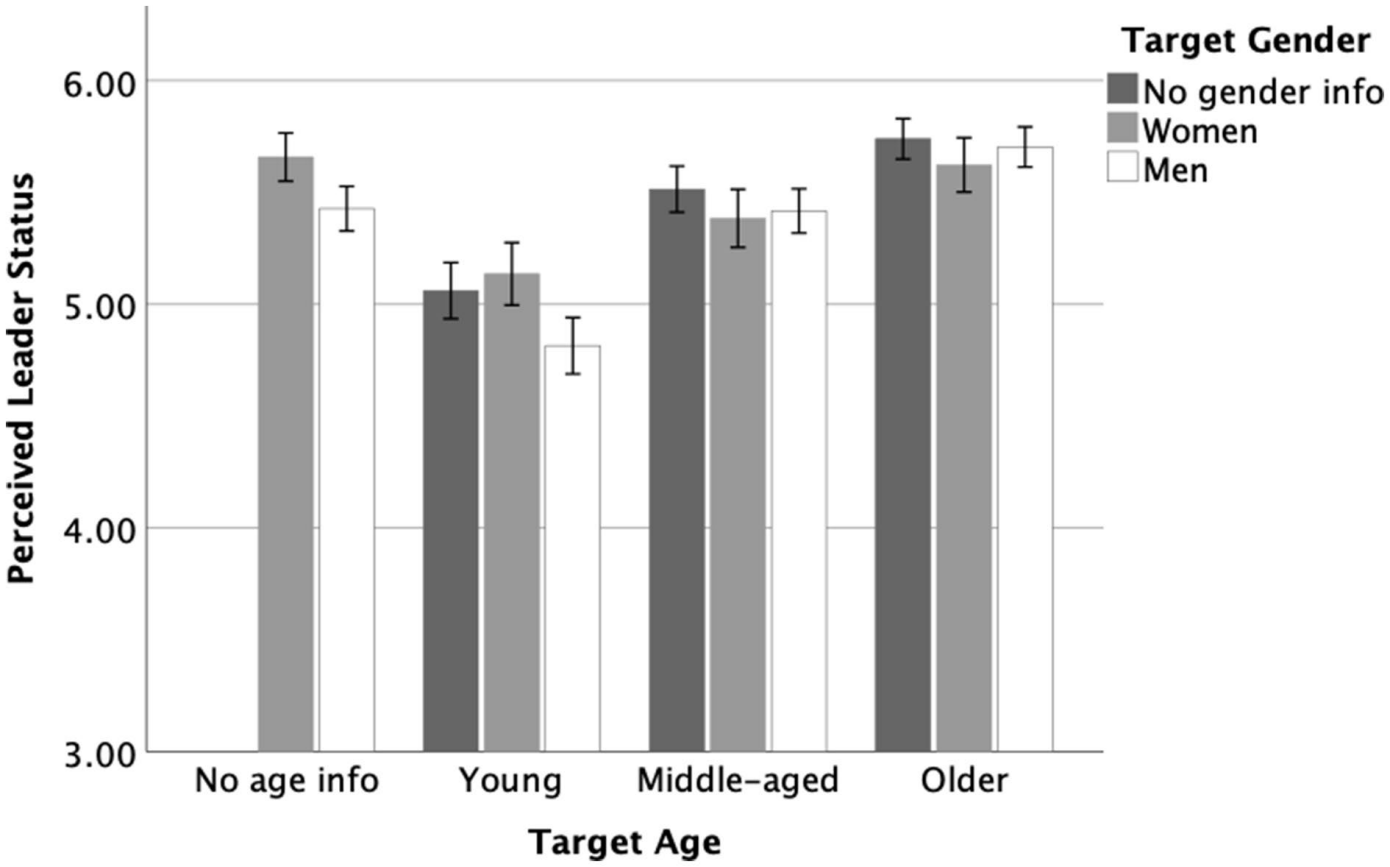
Study 2: Results for perceived leader status by target groups. Error bars represent standard errors.

In line with Hypothesis 2, young men were perceived to possess lower leader status than men in general, *t*(173) = −3.84, *p* < 0.001, *d* = −0.58, *M_diff_* = −0.61, 95% CI [−0.93, −0.30]. Additionally, there was no difference in perceived leader status between young men and young individuals, *t*(175) = −1.39, *p* = 0.168, *d* = −0.21, *M_diff_* = −0.25, 95% CI [−0.60, 0.10]. Figure 2 displays the ratings of perceived leader status for the different target groups.

Finally, following our approach in Study 1, we also compared the intersectional categories of young women and young men to identify differences *within* the young adult age group. In contrast to Study 1, the difference was not significant, although the direction of the effect showed that young women received slightly higher leader status ratings than young men, *t*(173) = 1.70, *p* = 0.09, *d* = 0.26, *M_diff_* = 0.32, 95% CI [−0.05, 0.69].

#### 5.2.4. Additional analyses

##### 5.2.4.1. Intersections of middle age and older age with gender

In addition to our hypothesis tests, we examined the intersectional effects of middle age and older age with gender on perceptions of leader status. This allows examining the alternative explanation that biased perceptions of leader status may not be specific to the intersection of young age with gender but instead be linked to the presence of age information more generally. To test this alternative explanation, we compare the perceived leader status of middle-aged and older women to women (*RQ1a/b*) and middle-aged and older men to men (*RQ2a/b*), respectively. To support the alternative explanation, we should find similar biased perceptions for the intersectional categories of middle-aged/older women and middle-aged/older men as we observed for young women or young men.

The results of independent samples *t*-tests showed no difference in perceived leader status for middle-aged women compared to women in general (*RQ1a*), *t*(180) = −1.63, *p* = 0.105, *d* = 0.24, *M_diff_* = −0.27, 95% CI [−0.61, 0.06]. Similarly, there was no difference in perceived leader status for older women compared to women in general (*RQ1b*), *t*(180) = −0.22, *p* = 0.827, *d* = 0.03, *M_diff_* = −0.04, 95% CI [−0.35, 0.28].

Comparing middle-aged and older men to men in general, the results showed no difference in perceived leader status between middle-aged men and men in general (*RQ2a*), *t*(177) = −0.07, *p* = 0.942, *d* = 0.01, *M_diff_* = −0.01, 95% CI [−0.29, 0.27] and a difference in perceived leader status for older men compared to men in general (*RQ2b*), with higher scores for older men, *t*(177) = 2.06, *p* = 0.04, *d* = 0.31, *M_diff_* = 0.28, 95% CI [0.01, 0.54]. These results do not support the alternative explanation that simply mentioning age information results in more negative perceptions of leaders.

Additionally, we compared the intersectional categories of middle-aged or older women and men within their own age groups. There were no significant differences in leader status ratings between middle-aged women and middle-aged men, *t*(178) = −0.20, *p* = 0.838, *d* = −0.03, *M_diff_* = −0.03, 95% CI [−0.36, 0.29]. Similarly, there were no significant differences in leader status ratings between older women and older men, *t*(178) = −0.53, *p* = 0.594, *d* = −0.08, *M_diff_* = −0.08, 95% CI [−0.38, 0.22].

### 5.3. Discussion (Study 2)

The findings from Study 2 largely replicate those from Study 1, with two exceptions. We did not find support for Hypothesis *H1a*, as there was no significant difference in perceived leader status between young women and young adults. However, young women were perceived to have lower leader status than women, which supports Hypothesis *H1b*. In line with Hypothesis *H2*, young men were seen as having lower leader status than men, a finding not supported in Study 1. No significant difference was observed between young men and young adults in terms of perceived leader status. In sum, young women were perceived as having lower status than women in general, whereas young men were perceived as having lower status than men in general. Both young women and young men did not differ from young adults in terms of perceived leader status.

Extending Study 1, our findings also supported Hypotheses *H3a* and *H3b*, indicating that leader status is perceived to be lower for young adults compared to middle-aged adults (*H3a*) and older adults (*H3b*). There was no difference between middle-aged and older adults. Our findings regarding the intersectional lenses of middle-aged and older age with gender (*RQ1a/b* and *RQ2a/b*) further revealed that the bias in leader perception is specific to the intersectional lens of *young* age and gender (see *H1b* and *H2*) and not broadly linked to the presence of age information. Contrary to Study 1, in which women were assigned higher leader status than men, we found no difference in perceived leader status between men and women when no age information was presented, even though the direction of findings was the same.

## 6. General discussion

The primary goal of this research was to develop a gender-specific understanding of the age bias toward young leaders. To examine the age bias toward young female and young male leaders, we integrated the lens-based account of intersectional stereotyping (Petsko et al., 2022) with status ascriptions based on age and gender (status characteristics theory; Berger et al., 1977; Ridgeway et al., 1985). Our findings from comparing various age groups reveal a strong explicit age bias against young leaders compared to middle-aged and older leaders.

The age bias against young leaders prevails even when gender information is considered. Our results show a similar pattern of bias affecting both young women and young men regarding perceived leader status. In particular, the intersectional lenses (i.e., young women or young men) lead to a more negative perception of leader status than the gender lenses (i.e., women or men). However, at least in Study 2, no differences were found between the intersectional and age lenses (young adults), suggesting that the intersectional bias is driven by age.

Notably, this bias pattern does not extend to middle-aged or older women and men, bolstering insights into a specific bias against young leaders. Our findings suggest that age plays a major role in leader status perceptions for young women and young men, while it appears to have a minor impact on leader status perceptions of middle-aged and older women or men. These results from Study 2 rule out the alternative explanation that providing age information in general, irrespective of the leader’s age, results in more negative evaluations.

### 6.1. Theoretical implications

Our results have valuable implications. First, there has been a debate about whether bias against members of intersectional group categories is additive or non-additive (e.g., Berdahl and Moore, 2006; Purdie-Vaughns and Eibach, 2008). We hypothesized that the intersectional lens (i.e., young women, young men) elicits status ascriptions that are not simply the average of the singular lenses (young adults, women, or men, respectively). These assumptions were supported for young men. Our findings reveal that the intersectional bias for young men is not simply the algebraic average of biases toward young adults and men since the lowest score is observed for young men (even though previous findings suggest that women should experience more bias than men). This suggests that age bias and gender bias interact in a non-additive manner.

The intersectional bias can be best explained by group prototypicality, as biases become amplified toward a group’s most prototypical member (Hall et al., 2019). Stereotypes associated with young adults (e.g., ambitious, self-directed; Francioli and North, 2021) and men (e.g., assertive, agentic; Eagly et al., 2020) may overlap, resulting in a more amplified age bias toward the intersectional category of young men (compared to young women). We found suggestive evidence for the group prototypicality of young men for young adults and young women for women (evident in stereotypical attribute associations such as *dominance*, see section #2.3.4 in the Supplementary material). Contrary to some of our assumptions, it seems that young women face age *and* gender status ascriptions, resulting in an algebraic mean for the intersectional category of young women. However, the differences between young women and young adults (Hypothesis *H1a*) are no longer significant in Study 2, indicating that the results for young women are less clear-cut.

Second, the age bias appears to persist when using an intersectional lens as it shows for both young women and young men. Interestingly, there is a difference in perceived leader status between young women and young men, with young women receiving higher scores in Study 1. However, these differences seem primarily driven by a gender effect, as the patterns in leader perception for women versus men and young women versus young men are quite similar. This suggests that the intersectional bias toward young men and young women is driven by young age. Further, in Study 2, we investigated whether the intersectional effects were specific to young individuals or generalize to middle-aged and older men and women. The findings indicate that biased perceptions of leader status are specific to the intersection of young age and gender, and not broadly associated with the presence of age information. Besides, the effects of gender appear to be more pronounced for young adults than for middle-aged and older adults. As age increases, the leader status perception differences between women and men diminish (i.e., the differences between middle-aged women and middle-aged men or older women and older men are smaller than those between young women and young men).

Third, our results build upon earlier studies showing that young adults in leadership positions are often negatively evaluated (e.g., Buengeler et al., 2016; Kunze and Menges, 2016). In line with our pre-registered hypotheses, we found an age bias specifically targeted at young leaders, as similar biases apparent in lower perceived leader status were not apparent for middle-aged and older leaders. Further, this pattern is consistent across different leadership dimensions, as we observed similar results for perceived leader effectiveness and liking compared to middle-aged leaders (yet not compared to older leaders; see Supplementary material sections #2.3.2 and #4.1.1). Hence, our data also suggest an age bias toward older leaders. Older leaders seemed to be perceived as less effective and likable than middle-aged leaders, although they were not ascribed lower status. These findings tentatively suggest that middle-aged adults may serve as a baseline standard regarding age in the workplace (e.g., Finkelstein et al., 2013), specifically in leadership positions.

Fourth, we did not find support for the expected gender bias against female leaders compared to male leaders. Conversely, compared to male leaders, female leaders were perceived as having more status (Study 1) or equal status (Study 2). One possible explanation is that without a direct threat, such as limited leadership positions, there might be no bias against groups based on static status characteristics such as gender or race (e.g., Kim et al., 2022). Another potential explanation for our findings might stem from participants’ awareness of prevalent research and expectations regarding gender stereotypes and bias. This could have led to socially desirable responses in their direct (explicit) leader ratings. We should note that the detection of gender bias can be nuanced, and while we employed direct measures in our studies, such explicit methods might not always fully expose these biases. Therefore, future research could benefit from implementing more indirect (implicit) methodologies akin to those used for uncovering racial bias in leadership studies (e.g., Petsko and Rosette, 2023).

Finally, young age seems to have a stronger impact than gender in leadership roles, at least when bias is assessed directly. This age bias in leadership supports previous research that shows people are likely to endorse age discrimination but not gender discrimination, even if people reject group-based hierarchies (i.e., people with egalitarian views; Martin and North, 2022). The age bias toward young adults also generalizes beyond status ascriptions, as revealed in perceived lower effectiveness and likeability (see Supplementary material sections #2.3.2 and #4.1.1). These encompassing negative evaluations of young leaders are unlikely to be solely due to their (perceived) lack of experience due to their age. While experience is often considered a proxy for competence, it should have less impact on likeability. These additional findings rather suggest an age bias against young leaders based on young age as a diffuse characteristics and therefore independent of an individual’s ability and expertise (Lianidou and Zheng, 2022).

### 6.2. Practical implications

Our results are relevant for optimizing human resource practices in organizations, including recruitment, selection, and performance evaluation. Whereas some studies found that the impact of age bias and stereotypes on personnel decisions is weak, nonexistent, or inconsistent (Murphy and DeNisi, 2022), other research demonstrates that age stereotypes influence personnel decisions throughout an employee’s career (Cadiz et al., 2022). We assume that even small differential treatments resulting from stereotypes can lead to severe consequences in binary decisions (e.g., promotion or dismissal; threshold models of behavior; Hester et al., 2020) or those that generate cumulative (dis)advantages, such as compensation and pay. Rather than focusing on young individuals’ actual competencies, expertise, and relevant characteristics for leadership roles (e.g., education, functional background, or specific status characteristics; Lianidou and Zheng, 2022), the lower ascribed status based on the diffuse status characteristic of young age may hinder young individuals from accessing leadership positions, cause biased evaluations, and may lead to a greater chance of dismissal for young leaders. Organizations should account for the age bias in leadership perception by adopting policies and practices that promote age diversity (e.g., Boehm et al., 2014) and incorporate diversity training programs (Homan et al., 2015).

### 6.3. Limitations and pertinent future research

Our findings stimulate several questions for future research. We focus on age-gender intersectionality while keeping other group categories unspecified (e.g., race). We intentionally used written text for the target group manipulation (e.g., female person, younger female person) rather than images of target group faces (e.g., Spisak et al., 2014), as images could prompt participants to apply a race or intersectional lens containing racial stereotypes, which our research does not address. Considering the importance of race and intersectional categories containing race in leadership perception (e.g., Petsko and Rosette, 2023), future research could address the intersections of young age and race in terms of group prototypicality (Hall et al., 2019) and leadership perception.

In our experimental approach, we asked participants to imagine a young person as their leader. This may add an extra layer of introspection, potentially complicating the evaluation process (i.e., “what it might mean or feel to be managed by a leader younger than oneself”). The impact of additional introspection on evaluations is expected to result in an interaction between evaluator age and leader ratings for young targets, as evaluators’ introspective processes, influenced by their own age, may alter their assessments of younger leaders. We tested the interaction of evaluator age on the target group and leader ratings in Study 1 (reported in the Supplementary material, section #2.3.1). However, apart from two exceptions (i.e., leader liking for young women/men compared to women), no interaction effects were identified, indicating limited additional introspection. Future studies could further explore age-inverse leadership relationships, particularly focusing on young leader age in both absolute and relative terms (e.g., Collins et al., 2009; Kunze and Menges, 2016).

## 7. Conclusion

In this manuscript, we develop a gender-specific understanding of age bias toward young leaders. We uncover an explicit negative age bias toward young leaders when compared to middle-aged and older leaders, a bias which persists across gender (i.e., young female and young male leaders). We examine the intersectional bias toward young female and male leaders by applying the lens-based account of intersectional stereotyping (Petsko et al., 2022). The intersectional lens yields a more negative perception of leader status than the gender lens (i.e., female and male leaders) with no differences between intersectional and age lenses (i.e., young leaders). Moreover, age has more influence, and gender less, for *young* leaders, possibly due to age’s less static nature as a status characteristic. Our research emphasizes the importance of considering evaluators’ lenses and demonstrates the negative impact of age bias on young leaders.

## Data availability statement

The dataset presented in Study 1 can be found at https://doi.org/10.23668/psycharchives.8236. The dataset presented in Study 2 can be found at https://osf.io/gmqt9/?view_only=81b8ac4b5f684d34a311a1c663bfad11.

## Ethics statement

Both studies received approval from the University of Amsterdam’s Economics & Business Ethics Committee, with protocol numbers EC 20220209020230 for Study 1 and EB-1013 for Study 2. In both studies, participants provided their informed consent to participate.

## Author contributions

CD: conceptualization, methodology, data collection, analyses, writing, and editing. CB and AH: conceptualization, methodology, writing, and editing. All authors contributed to the article and approved the submitted version.

## Funding

The Leibniz Institute for Psychology (ZPID) funded the data collection of Study 1. This work was also supported by the Joachim Herz Foundation through the Add-on Fellowship for Interdisciplinary Economics and Interdisciplinary Business Administration, awarded to Christoph Daldrop. Further, we acknowledge financial support from Land Schleswig-Holstein through the Open Access Publications Fund, which supported the open access publication process.

## Conflict of interest

The authors declare that the research was conducted in the absence of any commercial or financial relationships that could be construed as a potential conflict of interest.

## Publisher’s note

All claims expressed in this article are solely those of the authors and do not necessarily represent those of their affiliated organizations, or those of the publisher, the editors and the reviewers. Any product that may be evaluated in this article, or claim that may be made by its manufacturer, is not guaranteed or endorsed by the publisher.

## Footnotes

^1^In line with the gender-based hierarchy (Berger et al., 1977; Ridgeway, 2001) and the “men as the standard-paradigm” (e.g., Bailey et al., 2019), status ascriptions based on gender should be lower for women and non-binary individuals compared to men.

^2^In response to comments of the reviewers, we changed the wording, but not the content, of our pre-registered hypotheses in the paper. More specifically, we reformulated pre-registered Hypothesis 1 as current Hypothesis 2, reformulated and integrated pre-registered Hypotheses 3a and 3b into current Hypothesis 1a/b and moved a pre-registered moderation Hypothesis 2 to the supplement (more information about the predicted moderation of social dominance orientation is presented below in section 3).

^3^https://doi.org/10.23668/psycharchives.5404

^4^https://aspredicted.org/29S_GV2

^5^https://leibniz-psychology.org/en/services/data-collection/psychlab-online/

^6^https://www.respondi.com/

^7^We set the lower age boundary for young adults at 25, as most individuals have entered the workforce by this age. Following the US Discrimination in Employment Act of 1967 (ADEA), we established 39 as the upper boundary for young adults, reflecting those still in the early stages of their careers. Based on the OECD’s Directorate for Employment, Labour and Social Affairs, we defined older age in the workplace as beginning at 55. Consequently, we categorized middle-aged adults as those between 40 and 54 years old and older adults as those between 55 and 69, approaching the U.S. retirement age.

^8^In the U.S. population, 20.5% are between 25 and 39 years, 18.6% are between 40 and 54 years, and 18.2% are between 55 and 69 years (U.S. Census Bureau, 2021). As young adults (compared to middle-aged and older adults) are only marginally overrepresented in the actual composition of the U.S. population aged 25–69 years, we believe our approach of using similar sample sizes for the three age groups is acceptable.

^9^Following a reviewer’s recommendation, we conducted and reported independent samples *t*-tests in the main text, while offering the pre-registered regression analyses in the Supplementary material in section #2.2. This approach ensures transparency while easing interpretation. The findings and conclusions are the same.

^10^https://connect.cloudresearch.com
