# Supplementary material for: An intersectional lens on young leaders: bias toward young women and young men in leadership positions
Source: Front Psychol. 2023 Aug 16;14:1204547. doi: 10.3389/fpsyg.2023.1204547 (PMC10468608; doi:10.3389/fpsyg.2023.1204547)
Supplement: Supplementary file 1 [file Data_Sheet_1.pdf]

Supplementary Material

An Intersectional Lens on Young Leaders: Bias toward Young Women and Young Men in Leadership Positions

Christoph Daldrop*, Claudia Buengeler, and Astrid C. Homan

* **Correspondence:** Corresponding Author: daldrop@bwl.uni-kiel.de

Study 1 (Intersection of Young Age and Gender) 3
1 Method 3
1.1 Measures 3
1.1.1 Perceived Leader Effectiveness 3
1.1.2 Leader Liking 3
1.1.3 Social Dominance Orientation 3
1.1.4 Political Ideology (i.e., Right-wing authoritarianism scale) 4
1.1.5 Stereotypical Attributes 4
2 Results 4
2.1 Descriptive Statistics 4
2.2 Pre-registered Analyses Study 1 6
2.2.1 Bias toward Young Female Leaders 6
2.2.2 Bias toward Young Male Leaders 6
2.2.3 The Role of Social Dominance Orientation in Study 1 7
2.3 Exploratory Analyses Study 1 9
2.3.1 The Role of Evaluator Age in Leadership Perception 9
2.3.2 Perceived Leader Effectiveness and Leader Liking 12
2.3.3 The Mediating Role of Prescriptive Stereotypes in Study 1 14
2.3.4 Group Prototypicality of Young Women and Young Men in Study 1 16
Study 2 (Intersection of Age and Gender) 18
3 Method 18
3.1 Measures 18
4 Results 18
4.1 Exploratory Analyses 18
4.1.1 Perceived Leader Effectiveness and Leader Liking 18
4.1.2 The Role of Social Dominance Orientation in Study 2 20
5 References 23

Study 1 (Intersection of Young Age and Gender)

In the following, we report deviations from the pre-registration of Study 1 (see section 2.2). The pre-registration file can be accessed on OSF using this link: https://osf.io/gmqt9/?view_only=81b8ac4b5f684d34a311a1c663bfad11. We conducted hypotheses tests in sections 2.2.1 and 2.2.2 using regression analyses according to our pre-registered analysis plan. The results in the main manuscript (i.e., independent samples *t*-tests) were consistent with the pre-registered analyses in the supplemental materials (i.e., linear regression). To improve the readability and flow of our manuscript while maintaining the integrity of the pre-registered hypothesis, we relocated Hypothesis *H4* (i.e., the moderation of social dominance orientation on perceived leader status for young men compared to men; originally Hypothesis *H2* in the pre-registration) to the online supplements (see sections 2.2.3 and 4.1.2), as suggested by one anonymous reviewer.

Further, we present exploratory analyses based on reviewer comments and research questions pre-registered as exploratory analyses in section 2.3. In section 2.3.1, we present exploratory analyses regarding an additional layer of introspection, the role of evaluator age. Additionally, we moved research questions *RQ3a/b* and *RQ4* (i.e., perceived leader effectiveness and leader liking; originally labeled *E1* and *E2* in the pre-registration; see section 2.3.2), *RQ5a/b* (i.e., the mediating role of prescriptive stereotypes for leader status ascriptions; originally labeled *E3* in the pre-registration; see section 2.3.3), and *RQ6* (group prototypicality of young women and young men; originally labeled *E4* in the pre-registration; see section 2.3.4) that were pre-registered as exploratory analyses, to the online supplements due to reviewer suggestions.

1 Method

1.1 Measures

1.1.1 Perceived Leader Effectiveness

To assess perceived leader effectiveness, participants responded to three items used in prior research (e.g., Giessner & van Knippenberg, 2008; Gündemir et al., 2019), including "*This leader is a good leader*," "*This leader is effective as a leader*," and "*This leader is successful as a leader*." Participants used a 7-point scale to indicate perceived leader effectiveness (1 = *strongly disagree* to 7 = *strongly agree*).

1.1.2 Leader Liking

We measured leader liking by asking participants to respond to three items used in previous research (Rudman et al., 2012): "*How much do you like this leader?*", "*Is this leader someone you want to get to know better?*" and "*Would this leader be popular with colleagues?*". Participants used a 7-point scale to indicate perceived leader liking (1 = not at all to 7 = very much).

1.1.3 Social Dominance Orientation

To measure social dominance orientation (SDO), we used the eight items SDO_7 short scale (Ho et al., 2015) with the subdimensions of SDO-Anti-Egalitarianism (SDO-AE) and SDO-Dominance (SDO-D). An example item for SDO-D is "s*ome groups of people are simply inferior to other groups*." An example item for SDO-E is "*group equality should not be our primary goal*." Participants used a 7-

point scale to indicate social dominance orientation (1 = *strongly oppose* to *7* = *strongly favor*). To ensure that all items were consistent in terms of agreement or disagreement, we first re-coded the responses of the four reverse-coded items so that a high score is transformed into the corresponding low score and vice versa (for example, in a 7-point scale, a *7* is transformed into a *1*). We calculated a single "social dominance orientation" variable by computing the mean for the eight items.

1.1.4 Political Ideology (i.e., Right-wing authoritarianism scale)

We measured right-wing authoritarianism (RWA) with the six items "balanced short scale of authoritarian attitudes" (B-RWA-6; Aichholzer & Zeglovits, 2015). Right-wing authoritarianism is described by three covarying attitudinal clusters: authoritarian submission, authoritarian aggression, and conventionalism. An example item for submission is "*We should be grateful for leaders that tell us exactly what we shall do and how*." An example item for aggression is "*our society for once has to crack down harder on criminals*." An example item for conventionalism is "*This country would flourish if young people paid more attention to traditions and values* ." Participants use a 7-point scale to indicate right-wing authoritarianism (1 = *strongly oppose* to *7* = *strongly favor*). To ensure that all items are consistent in terms of agreement or disagreement, we first re-coded the responses of the three reverse-coded items so that for all items of the scale, a high score indicated more right-wing authoritarianism. We calculated a single "right-wing authoritarianism" variable by computing the mean for the six items.

1.1.5 Stereotypical Attributes

We included attributes associated most with age, gender, and leader roles: dominance, competence, friendliness, and morality (Abele et al., 2021). We used the 46-item scale by Offermann and Coats (2018) as this is the most recent leader attribute scale and includes the stereotype dimensions of dominance (15 items, e.g., *strong*, *authoritative*), competence (11 items, e.g., *intelligent*, *dedicated*), and friendliness (8 items, e.g., *sensitive*, *kind*). We added two additional attributes (*dominant, competent*) that indicated dominance and competence and were not part of the leader attribute scale by Offermann and Coats (2018). We also included six attributes to measure the stereotype dimension of morality (e.g., *trustworthy*, *moral*; Abele et al., 2016). Participants used a 5-point scale to indicate the extent to which each attribute is undesirable (i.e., proscriptions) or desirable (i.e., prescriptions) of the specific target group (1 = *undesirable*, 2 = *somewhat undesirable*, 3 = *neutral*, 4 = *somewhat desirable*, 5 = *desirable*). This approach is similar to the prescriptive stereotype question and response options used in Prentice and Carranza (2002) bipolar scale. We presented the series of attributes in six blocks of nine attributes each (blocks and attributes within each block randomized). Reliabilities are presented in Table S1.

2 Results

2.1 Descriptive Statistics

We report correlations among the study variables, including demographics (evaluator age, evaluator gender, RWA, SDO), independent variables (dummy variables for the target groups), stereotypes separated for the different dimensions (dominance, competence, friendliness, morality), and main dependent variables (perceived leader status, perceived leader effectiveness, leader liking) in Table S1.

Table S1. Correlations Between Study Variables in Study 1

	1.	2.	3.	4.	5.	6.	7.	8.	9.	10.	11.	12.	13.	14.	15.	16.
1. Evaluator Age	--															
2. Evaluator Gender[a]	.01	--														
3. Social Dominance Orientation	.00	.02	(.83)													
4. Right-wing Authoritarianism	.15**	.01	.34**	(.52)												
5. Young Adults[b]	.06	.04	.00	-.08*	--											
6. Young Men[b]	-.03	-.05	-.01	.06	-.24**	--										
7. Men[b]	-.02	.01	.03	.04	-.26**	-.25**	--									
8. Young Women[b]	.00	-.01	-.01	.00	-.24**	-.23**	-.25**	--								
9. Women[b]	-.01	.00	-.01	-.02	-.26**	-.25**	-.28**	-.25**	--							
10. Perceived Leader Status	-.03	.02	-.15**	.05	-.12**	-.07*	.01	.03	.15**	(.93)						
11. Perceived Leader Effectiveness	.02	-.03	-.18**	.10**	-.12**	-.04	-.03	.03	.15**	.70**	(.97)					
12. Leader Liking	-.04	-.02	-.15**	.08*	-.07*	-.06	-.04	.03	.15**	.65**	.70**	(.88)				
13. Dominance	-.22**	.12**	.13**	.01	-.14**	-.11**	.02	.08*	.14**	.19**	.15**	.24**	(.87)			
14. Competence	.02	-.02	-.23**	.05	-.05	.02	.03	-.03	.02	.39**	.36**	.32**	.08*	(.90)		
15. Friendliness	.07*	-.07	-.21**	.06	.00	.01	.09**	-.06	-.04	.30**	.29**	.32**	-.09**	.52**	(.85)	
16. Morality	.17**	-.04	-.25**	.05	-.03	.04	.06	-.04	-.03	.28**	.29**	.25**	-.16**	.70**	.65**	(.81)

Note. $N = 918$. [a] Female coded 0, Male coded 1. [b] Each dummy variable groups one target condition (e.g., young targets coded 1) against the other four target conditions (e.g., young men, men, young women, and women coded 0) and therefore provides only limited information regarding bivariate correlations. Including four dummies in the regression analyses is more informative regarding the relationships between two target conditions. Reliabilities of scales are listed in parentheses on the diagonal.

*$p < .05$; **$p < .01$

2.2 Pre-registered Analyses Study 1

2.2.1 Bias toward Young Female Leaders

We conducted linear regression analyses using IBM SPSS 29 to test our hypotheses aligned with our pre-registered analyses plan. The dependent variable for Hypotheses *H1a* and *H1b* was perceived leader status. We created four dummy variables representing five conditions. The reference category was the young women condition (i.e., young female targets scored 0 on all dummy variables), which was compared against the following conditions: young individuals (i.e., young targets coded as -1), women (i.e., female targets coded as -1), young men (i.e., young male targets coded as -1), and men (i.e., male targets coded as -1).

The results revealed that young women were perceived to have a higher leader status than young individuals, supporting Hypothesis *H1a*. In contrast, young women were perceived to have a lower leader status than women, supporting Hypothesis *H1b* (see Table S2). Additionally, young women were perceived as having a higher leader status than young men, while no significant differences were found between young women and men.

Table S2. Results of Regression Analysis for Perceived Leader Status of Young Women Compared to Other Group Categories in Study 1

		95% CI		
	β	LL	UL	*p*
**Young Women vs. Young (*H1a*)**	**.12**	**.05**	**.24**	**.00**
**Young Women vs. Women (*H1b*)**	**-.09**	**-.20**	**-.01**	**.03**
Young Women vs. Young Men	.08	.00	.19	.04
Young Women vs. Men	.02	-.08	.12	.66
R2	.04			
F	8.26			

Note. *N* =918. Standardized coefficients (*β*s) and 95% confidence intervals are reported; LL = lower limit; UL = upper limit. Hypotheses tests are reported in bold.

2.2.2 Bias toward Young Male Leaders

To test Hypothesis *H2*, we performed a linear regression analysis with perceived leader status as the dependent variable. Again, we created four dummy variables to represent five conditions. In this case, the reference category was young men (i.e., young male targets scored 0 on all dummy variables), which was compared against the following conditions: young individuals (i.e., young targets coded as -1), men (i.e., male targets coded as -1), young women (i.e., young female targets coded as -1), and women (i.e., female targets coded as -1).

Using the pre-registered linear regression analysis, we found no significant difference in perceived leader status between young men and men (H2, see Table S3). These findings align with those presented in the main manuscript.

2.2.3 The Role of Social Dominance Orientation in Study 1

Theories on why individuals with subordinate group memberships face bias further stem from social dominance orientation research (Pratto et al., 1994; Sidanius & Pratto, 1999). Social dominance theory posits that status effects can be explained by people's support toward established social hierarchies. Research has shown that social dominance orientation predicts bias and prejudice, including sexism and racism (e.g., Levin et al., 2012; Sibley et al., 2007). Targets of bias are often the most visible members (i.e., the most prototypical members) of a subordinate group. Specifically, individuals with a preference for group-based hierarchy and inequality (i.e., higher social dominance orientation; Ho et al., 2015; Pratto et al., 1994) may perceive especially male members of a minority group as a threat (i.e., subordinate male target hypothesis; Sidanius & Pratto, 1999).

Applying the idea of a preference for group-based hierarchy to age and the age-based hierarchy, we propose that followers' social dominance orientation strengthens the bias toward young male leaders. Evaluators with a preference for group-based hierarchy and inequality (i.e., higher social dominance orientation; Ho et al., 2015; Pratto et al., 1994) should evaluate young male leaders more negatively. To test this assumption, we consider social dominance orientation as a contingency factor for the leadership perception of young men. Therefore, we propose the following:

> *H4: The bias in the form of lower perceived status toward young male leaders (compared to male leaders) is stronger for evaluators with higher social dominance orientation than evaluators with lower social dominance orientation.*[1]

To test the predicted interaction effect of social dominance orientation and young men on perceived leader status, we calculated the product of social dominance orientation and the four dummy variables. Not supporting Hypothesis *H4*, the interaction term "Young Men vs. Men x SDO" was not related to perceived leader status (see Table S3).

Additionally, we conducted an exploratory analysis comparing young individuals to men. The contrast of young individuals and men unique to this analysis is reported in Table S3. Young individuals were perceived to have less leader status than men, and social dominance orientation moderated this negative relationship. Simple slopes of social dominance orientation on perceived leader status, separated for the young versus men condition, are presented in Figure S1 (Aiken & West, 1991; McCabe et al., 2018). As revealed by the simple slope tests, social dominance orientation was significantly negatively related to perceived leader status for young targets, while it was non-significant for male targets.

---

[1] In line with comments from the reviewers, we changed the wording of our pre-registered hypotheses. Even though the meaning remains unchanged, there is now an inconsistency in the wording between the pre-registration file and the way hypotheses are presented in the Supplementary Material.

Table S3. Results of Regression Analysis for Perceived Leader Status of Young Men in Study 1

		95% CI				95% CI		
	β	LL	UL	*p*	*β*	LL	UL	*p*
Main Effects (Step 1)								
Young Men vs. Young	.04	-.05	.14	.39	.04	-.05	.14	.37
**Young Men vs. Men (*H2*)**	**-.08**	**-.19**	**.01**	**.07**	-.07	-.18	.01	.09
Young Men vs. Young Women	-.08	-.19	.00	.04	-.08	-.19	.00	.05
Young Men vs. Women	-.18	-.31	-.12	.00	-.18	-.31	-.12	.00
(Young vs. Men) [a]	-.11	-.23	-.04	.01	-.11	-.22	-.03	.01
SDO (centered)	-.15	-.25	-.10	.00	-.05	-.25	.12	.51
Two-way Interactions (Step 2)								
Young Men vs. Young x SDO					.09	.00	.21	.06
**Young Men vs. Men x SDO (*H4*)**					**-.01**	**-.13**	**.10**	**.78**
Young Men vs. Young Women x SDO					.08	-.02	.20	.10
Young Men vs. Women x SDO					.07	-.03	.20	.15
(Young vs. Men x SDO)[a]					-.12	-.25	-.02	.02
R2	.06				.07			
F	11.2				7.29			

Note. *N* = 918. Standardized coefficients (*βs*) are reported. CI = confidence interval; LL = lower limit; UL = upper limit; SDO = social dominance orientation. Hypotheses tests are reported in bold.
[a] We only report the contrast between the young targets and men unique to the additional analysis.

Figure S1. Simple Slopes for Perceived Leader Status by Social Dominance Orientation Separated by Target Group in Study 1

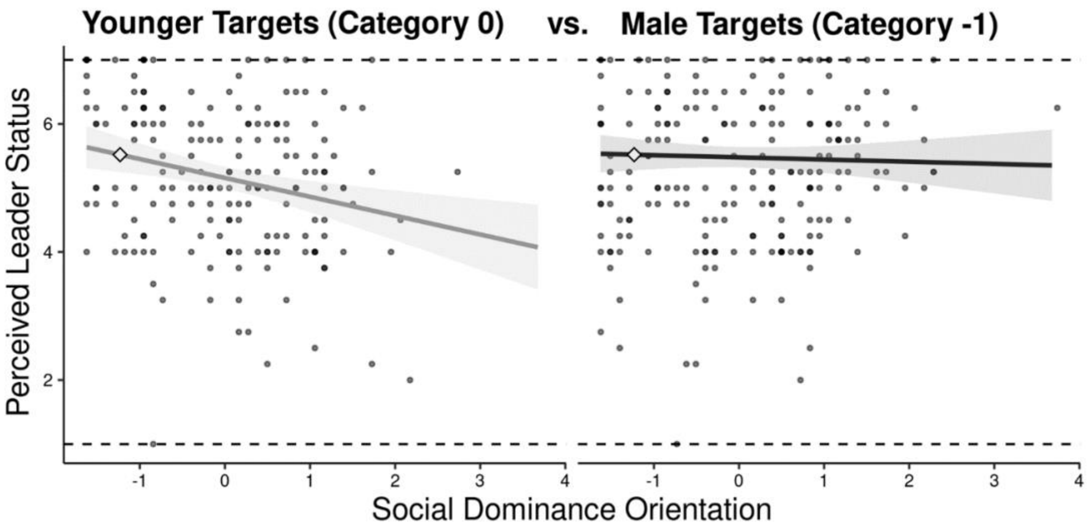

Note. The figure shows the computed 95% confidence region (shaded area), the observed data (gray circles), the maximum and minimum values of the outcome (dashed horizontal lines), and the crossover point (diamond).

2.3 Exploratory Analyses Study 1

2.3.1 The Role of Evaluator Age in Leadership Perception

In response to an anonymous reviewer's suggestion, we explored the role of *introspection*, specifically focusing on the potential implications and participants' feelings associated with being managed by a leader *younger* than themselves. Therefore, we tested the role of evaluator age in perceived leader status, effectiveness, and liking across different target groups. We conducted a linear regression analysis with perceived leader status as the dependent variable and, as before, created four dummy variables to represent five conditions. To assess the interaction effect with evaluator age, we calculated the product of the centered evaluator age and the four dummy variables.

We tentatively expected to find an interaction of evaluator age on leader ratings when comparing target conditions that include a young age group (i.e., young adults, young men, young women) with target groups without age information in the scenario (i.e., men, women). With only a few exceptions (i.e., leader liking for young women/men compared to women), we did not find that evaluator age moderated our effects. This suggests that introspection may not play a significant role in the evaluation of young adults, young men, or young women as participants' current leaders (see Tables S4 and S5).

Table S4. Results of Regression Analyses for Leadership Ratings of Young Women in Study 1

	Perceived Leader Status		Perceived Leader Effectiveness		Leader Liking	
	β [LL, UL]	*p*	*β* [LL, UL]	*p*	*β* [LL, UL]	*p*
Main Effects (Step 1)						
Young Women vs. Young	.13 [.05, .24]	<.01	.13 [.06, .26]	<.01	.08 [.00, .19]	.05
Young Women vs. Women	-.09 [-.20, -.01]	.03	-.09 [-.21, -.01]	.03	-.09 [-.20, -.01]	.03
Young Women vs. Young Men	.08 [.00, .19]	.05	.06 [-.02, .17]	.14	.08 [-.00, .19]	.06
Young Women vs. Men	.02 [-.07, .12]	.65	.05 [-.04, .16]	.24	.06 [-.02, .17]	.16
Evaluator age (centered)	-.18 [-.03, -.00]	.01	-.09 [-.02, .00]	.20	-.10 [-.02, .00]	.18
Two-way Interactions (Step 2)						
Young Women vs. Young x Evaluator Age	-.13 [-.26, -.04]	<.01	-.13 [-.27, -.04]	.01	-.06 [-.18, .03]	.16
Young Women vs. Women x Evaluator Age	-.08 [-.20, .01]	.06	-.06 [-.18, .04]	.19	-.10 [-.22, -.01]	.02
Young Women vs. Young Men x Evaluator Age	-.08 [-.20, .01]	.08	-.03 [-.14, .07]	.48	.01 [-.09, .12]	.77
Young Women vs. Men x Evaluator Age	-.05 [-.17, .04]	.23	-.03 [-.15, .07]	.46	.01 [-.09, .12]	.85
R2	0.05		0.04		0.03	
F	4.71		4.51		4.19	

Note. *N* =918. Standardized coefficients (*β*s) and 95% confidence intervals are reported; LL = lower limit; UL = upper limit. The young women condition was the condition of interest (i.e., young female targets score a 0 on all dummy variables) that was compared with the young individuals (i.e., young targets coded -1), women (i.e., female targets coded -1), young men (i.e., young male targets coded -1), and men (i.e., male targets coded -1).

Table S5. Results of Regression Analyses for Leadership Ratings of Young Men in Study 1

	Perceived Leader Status		Perceived Leader Effectiveness		Leader Liking	
	β [LL, UL]	*p*	*β* [LL, UL]	*p*	*β* [LL, UL]	*p*
Main Effects (Step 1)						
Young Men vs. Young	.04 [-.04, .15]	.31	.07 [-.01, .18]	.11	.00 [-.09, .10]	.93
Young Men vs. Men	-.06 [-.17, .02]	.10	-.01 [-.11, .08]	.71	-.02 [-.12, .07]	.58
Young Men vs. Young Women	-.08 [-.19, -.00]	.05	-.06 [-.17, .02]	.14	-.07 [-.18, .00]	.06
Young Men vs. Women	-.18 [-.30, -.11]	.00	-.15 [-.29, -.09]	.00	-.17 [-.30, -.10]	.00
Evaluator Age (centered)	-.00 [-.17, .17]	.97	-.02 [-.20, .15]	.76	-.13 [-.32, .01]	.07
Two-way Interactions (Step 2)						
Young Men vs. Young x Evaluator Age	-.05 [-.16, . 05]	.29	-.09 [-.23, -.00]	.04	-.08 [-.20, .01]	.08
Young Men vs. Men x Evaluator Age	.03 [-.07, .14]	.55	-.00 [-.11, .11]	.98	-.00 [-.11, .10]	.91
Young Men vs. Young Women x Evaluator Age	.08 [-.01, .20]	.08	.03 [-.06, .15]	.48	-.01 [-.11, .09]	.77
Young Men vs. Women x Evaluator Age	-.00 [-.11, .10]	.92	-.02 [.14, .08]	.55	-.11 [0.24, -.03]	.10
R2	0.05		0.04		0.03	
F	4.71		4.51		4.19	

Note. *N* =918. Standardized coefficients (*β*s) and 95% confidence interval are reported; LL = lower limit; UL = upper limit. Young men were the condition of interest (i.e., young male targets score a 0 on all four dummies) that were compared with young individuals (young targets coded -1), men (male targets coded -1), young women (i.e., young female targets coded -1), and women (i.e., female targets coded -1).

2.3.2 Perceived Leader Effectiveness and Leader Liking

Research has argued and found that lower-status beliefs are consequential regarding leader evaluations (e.g., perceived leader effectiveness) and follower or group outcomes (e.g., job satisfaction and turnover intentions; see Lianidou & Zheng, 2022 for a recent literature review). We argue that when a leader's diffuse status characteristics (e.g., age or gender) indicate a lower status, other leader ratings should also be negatively affected. To further test for bias toward young female and young male leaders, we examine whether ratings of leader effectiveness and liking are similarly influenced. Therefore, we propose the following research questions:

> *RQ3a/b: Are young female leaders perceived as (a) more effective and likable than young leaders in general but (b) less effective and likable than female leaders?*

> *RQ4: Are young male leaders perceived as less effective and likable than male leaders?*

Addressing research question *RQ3a/b*, our analysis demonstrates that young female leaders are perceived as more effective than young leaders in general but less effective than female leaders. Furthermore, young female leaders are not considered more likable than young leaders in general and are viewed as less likable than female leaders.

In response to research question *RQ4*, young male leaders are not significantly less effective or likable than male leaders. Additionally, they are perceived as similarly effective and likable compared to young leaders. We present the result in Table S6 and Figure S2.

Table S6. Means and Standard Deviations of Leader Ratings for Target Groups in Study 1

Perceived Leader Effectiveness	No Age Info	Young
No Gender Info		5.39^{b} (1.19)
Women	6.03^{a} (1.18)	5.76^{c} (1.15)
Men	5.62^{bc} (1.25)	5.57^{bc} (1.14)
**Leader Liking**	**No Age Info**	**Young**
No Gender Info		5.17^{b} (1.16)
Women	5.67^{a} (1.14)	5.41^{b} (1.15)
Men	5.24^{b} (1.12)	5.18^{b} (1.19)

Note. Standard deviations are reported in brackets. Means that share the same superscripts for each outcome variable did *not* differ at the $p < .05$ level.

Figure S2. Ratings of Perceived Leader Effectiveness and Leader Liking by Target Groups in Study 1

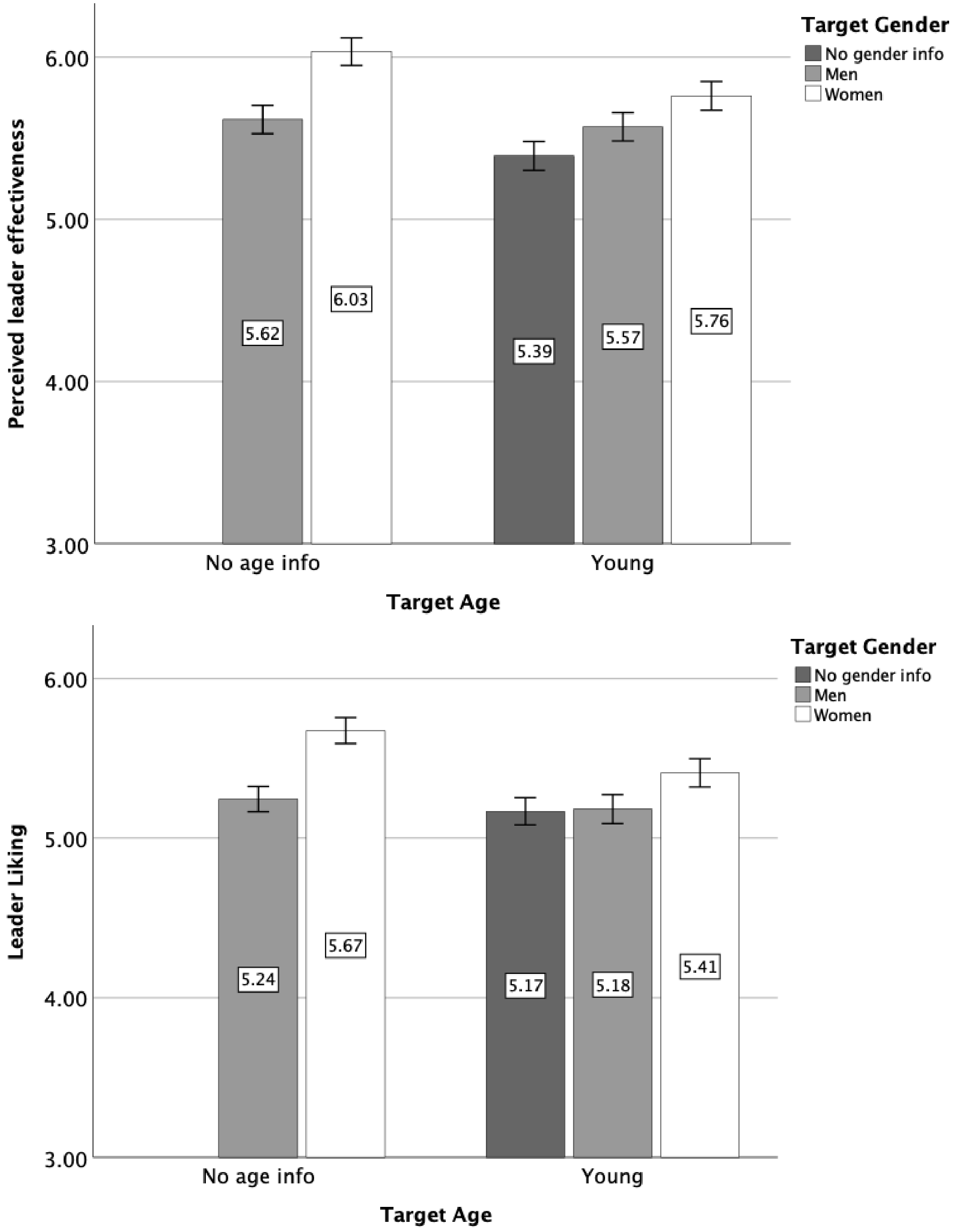

Note. Error bars represent standard errors.

2.3.3 The Mediating Role of Prescriptive Stereotypes in Study 1

To gain a deeper understanding of biases against young leaders, we examined the content of prescriptive stereotypes associated with age, gender, and intersectional group categories in an exploratory manner. We are particularly interested in the stereotypical attributes linked to leader status, specifically dominance and competence (Offermann & Coats, 2018). Our analyses focus on the relationship between prescriptive stereotypes and biases in leadership perception for young women and young men. As such, we propose the following research question:

> *RQ5a/b: Do the prescriptive stereotypes of dominance and competence mediate the bias in the form of lower perceived leader status for (a) young women and (b) young men?*

Given the main effect of young women compared to young individuals (*H1a*) and women (*H1b*) on perceived leader status in Study 1, we conducted an exploratory analysis to determine if these effects are associated with stereotypes of dominance and competence (pre-registered Research Question *RQ5a*). To estimate the indirect effect, we used the PROCESS macro (Model 4; Hayes, 2013) and calculated bias-corrected confidence intervals (CI) based on 5,000 bootstrap samples (Hayes, 2013; Preacher & Hayes, 2004). Our analyses indicated a positive indirect effect for young women compared to young individuals on perceived leader status (*H1a*) via dominance, indirect effect = .028, *SE* = .01, 95% CI [.012, .047], but not via competence, indirect effect = .008, *SE* = .02, 95% CI [-.031, .050]. The negative effect of young women compared to women on perceived leader status (*H1b*) could neither be linked to dominance, indirect effect = -.006, *SE* = .01, 95% CI [-.020, .007], nor competence stereotypes, indirect effect = -.019, *SE* = .02, 95% CI [-.058, .019]. We present results for the direct effects of the different target conditions on stereotypes of dominance, competence, and perceived leader status in Table S7.

We explored the possibility of an indirect effect through dominance and competence stereotypes (pre-registered Research Question *RQ5b*). Using the PROCESS macro (Model 4; Hayes, 2013), we discovered a negative indirect effect for young men (compared to men) on perceived leader status via dominance stereotypes, indirect effect = -.018, *SE* = .01, 95% CI [-.036, -.003]. Additionally, no indirect effect was observed for young men compared to men on perceived leader status via competence stereotypes, indirect effect = -.001, *SE* = .02, 95% CI [-.035, .033] (see Table S8).

Table S7. Results of Regression Analyses for Young Women in Study 1

	Dominance (prescriptive)				Competence (prescriptive)				Perceived Leader Status			
		95% CI				95% CI				95% CI		
	β	LL	UL	*p*	*β*	LL	UL	*p*	*β*	LL	UL	*p*
Main effects												
YW vs. Y	.18	.10	.26	<.01	.02	-.07	.10	.68	.11	.02	.20	.02
YW vs. W	-.04	-.12	.04	.35	-.04	-.13	.04	.31	-.08	-.17	.01	.07
YW vs. YM	.15	.07	.24	<.01	-.04	-.13	.04	.30	.09	.01	.18	.04
YW vs. M	.05	-.03	.13	.23	-.05	-.13	.04	.25	.04	-.05	.12	.44
Mediator												
Dominance									.15	.08	.22	<.01
Competence									.43	.36	.50	<.01
R2	.05				.01				.20			
F	10.97				1.05				37.06			

Note. *N* = 918. Standardized coefficients (*β*s) were reported. CI = confidence interval; LL = lower limit; UL = upper limit. YW = Young women, Y= Young adults, W = Women, YM = Young men, M = Men. Dominance and competence were centered.

Table S8. Results of Regression Analyses for Young Men in Study 1

	Dominance (prescriptive)				Competence (prescriptive)				Perceived Leader Status			
		95% CI				95% CI				95% CI		
	β	LL	UL	*P*	*β*	LL	UL	*p*	*β*	LL	UL	*p*
Main effects												
YM vs. Y	.02	-.06	.10	.61	.06	-.02	.15	.14	.01	-.07	.10	.76
YM vs. M	-.11	-.20	-.03	<.01	.00	-.08	-.08	.95	-.06	-.15	.03	.16
YM vs. YW	-.15	-.24	-.07	<.01	.04	-.04	.13	.30	-.09	-.18	-.01	.04
YM vs. W	-.20	-.28	-.12	<.01	.00	-.08	.09	.96	-.18	-.27	-.09	<.01
Mediator												
Dominance									.15	.08	.22	<.01
Competence									.43	.36	.50	<.01
R2	.05				.01				.20			
F	10.97				1.05				37.06			

Note. *N* = 918. Standardized coefficients (*β*s) were reported. CI = confidence interval; LL = lower limit; UL = upper limit. YM = Young men, Y= Young adults, M = Men, YW = Young women, W = Women. Dominance and competence were centered.

2.3.4 Group Prototypicality of Young Women and Young Men in Study 1

Our reasoning for Hypothesis *H1a* is that observers perceive young women as less prototypical members of the younger age group than young men. This is rooted in a gender-based hierarchy, implying that people tend to define men as the prototypical members of a given group (Bailey et al., 2019). Further, this rationale aligns with the assumption of the incongruity of prescriptive stereotypes toward women (e.g., less dominant) and young adults (e.g., more dominant). In contrast, there might be a congruence of prescriptive stereotypes toward men and young adults (e.g., more assertive). To test the concept of group prototypicality (Hall et al., 2019) in an exploratory way, we examine the following research question:

> *RQ6: Do observers perceive young women as less prototypical members of the younger age group than young men?*

We conducted an exploratory analysis of the age and gender prototypicality of young women and young men by comparing mean ratings of prescriptive stereotypes related to dominance, competence, friendliness, and morality (see Table S9). In line with our assumptions, young women shared more prescriptive stereotypes of dominance with women than with young adults. Further, participants held similar prescriptive stereotypes of dominance for young men and young adults (see Figure S3), whereas both differed from men in general. Additionally, there were no significant differences in stereotypes of friendliness for young adults, men, and young men. There were only small differences in the prescriptive stereotypes of competence and morality across all target groups.

Table S9. Means and Standard Deviations of Prescriptive Stereotypes for Target Groups in Study 1

**Dominance**	No age info	Young	**Friendliness**	No age info	Young
No gender info		2.43^{c} (0.52)	No gender info		4.16^{ab} (0.58)
Women	2.78^{a} (0.62)	2.72^{ab} (0.58)	Women	4.13^{a} (0.59)	4.09^{a} (0.57)
Men	2.64^{b} (0.72)	2.47^{c} (0.63)	Men	4.26^{b} (0.61)	4.18^{ab} (0.57)
**Competence**	No age info	Young	**Morality**	No age info	Young
No gender info		4.45^{a} (0.49)	No gender info		4.51^{ab} (0.47)
Women	4.53^{a} (0.50)	4.48^{a} (0.55)	Women	4.51^{ab} (0.55)	4.50^{b} (0.56)
Men	4.53^{a} (0.46)	4.53^{a} (0.42)	Men	4.60^{a} (0.49)	4.58^{ab} (0.45)

Note. Standard deviations are reported in brackets. Means that share the same superscripts for each outcome variable did *not* differ at the $p < .05$ level.

Figure S3. Ratings of Dominance by Target Groups in Study 1

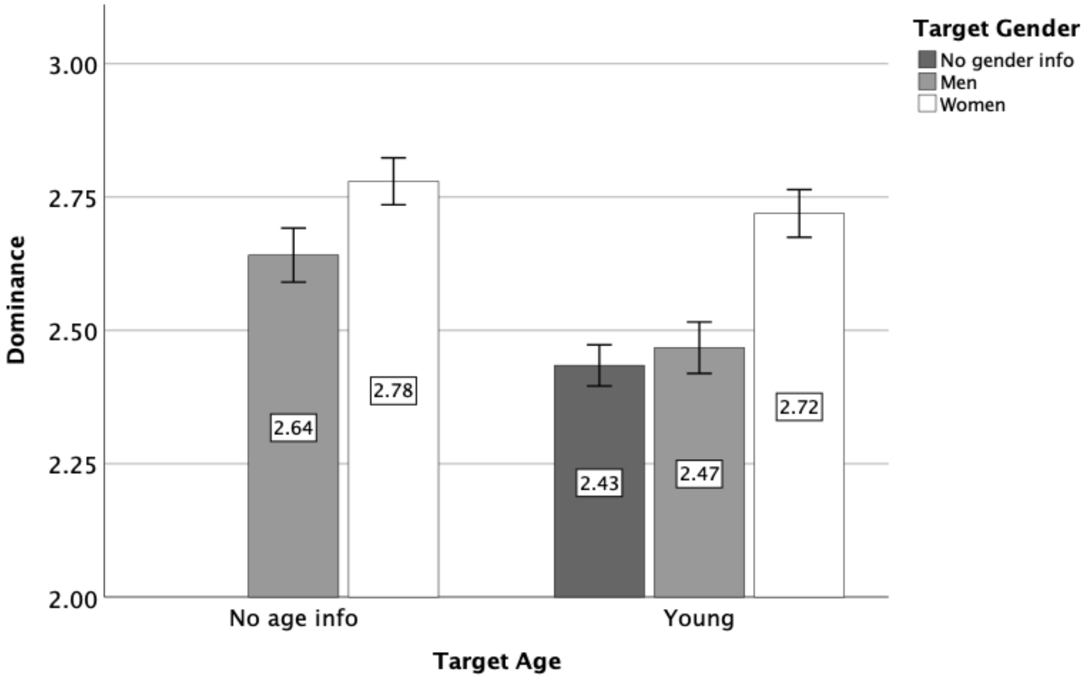

Note. Error bars represent standard errors.

Study 2 (Intersection of Age and Gender)

3 Method

3.1 Measures

We used the same leader evaluation measures as in Study 1. These measures include perceived leader effectiveness (e.g., Giessner & van Knippenberg, 2008; Gündemir et al., 2019) and leader liking (Rudman et al., 2012). Cronbach's alpha for perceived leader effectiveness was .96, while it was .85 for leader liking. Additionally, we measured social dominance orientation (SDO) with the same eight items from the SDO_7 short scale (Ho et al., 2015) as we did in Study 1. Cronbach's alpha for SDO was .91.

4 Results

4.1 Exploratory Analyses

4.1.1 Perceived Leader Effectiveness and Leader Liking

We present mean ratings and standard deviations of perceived leader effectiveness and leader liking for each target condition in Table S10. Figure S4 illustrates the ratings of perceived leader effectiveness and leader liking across the various target groups, with error bars representing standard errors.

Table S10. Means and Standard Deviations of Leader Ratings for Target Groups in Study 2

Perceived Leader Effectiveness	No age info	Young	Middle-aged	Older
No gender info		4.77 (1.22)	5.52 (1.06)	5.52 (0.95)
Women	5.58 (1.07)	5.16 (1.14)	5.43 (1.41)	5.53(1.07)
Men	5.19 (1.00)	4.83 (1.14)	5.24 (1.02)	5.39 (0.92)
**Leader Liking**	**No age info**	**Young**	**Middle-aged**	**Older**
No gender info		4.81 (1.13)	5.15 (1.14)	5.11 (1.09)
Women	5.32 (1.06)	5.17 (1.04)	5.26 (1.32)	5.17 (1.03)
Men	4.91 (1.02)	4.71 (1.06)	5.04 (0.98)	4.87 (1.00)

Note. N = 985. Standard Deviations are reported in brackets.

Figure S4. Ratings of Perceived Leader Effectiveness and Leader Liking by Target Groups in Study 2

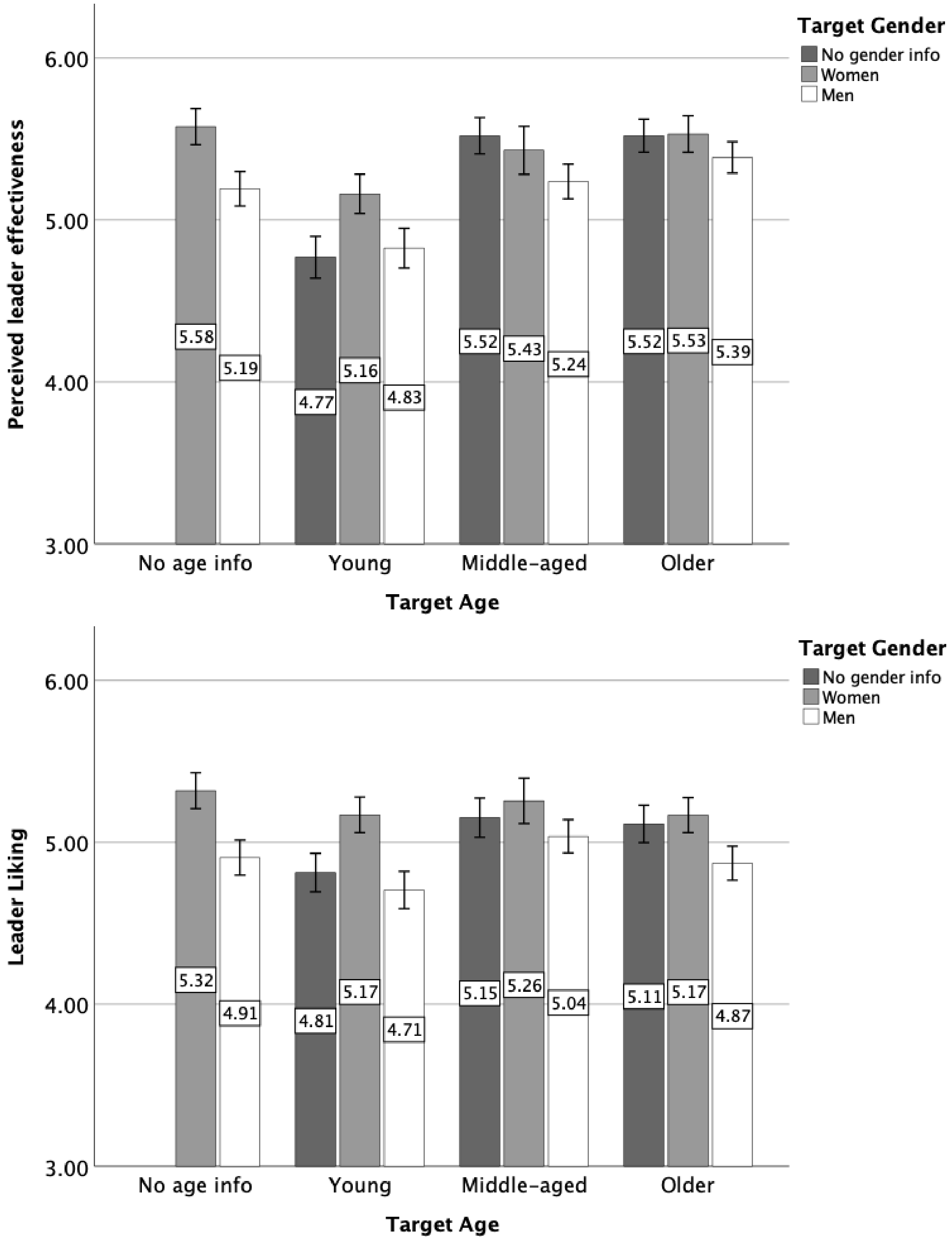

Note. Error bars represent standard errors.

4.1.2 The Role of Social Dominance Orientation in Study 2

In Study 2, we again tested our pre-registered Hypothesis *H4* similar to our analysis in Study 1. Not supporting Hypothesis *H4*, the interaction term "Young Men vs. Men x SDO" was not related to perceived leader status (see Table S11). Interestingly, we found a significant interaction effect of social dominance orientation and the comparison between young men and young women on perceived leader status. A higher social dominance orientation was related to lower perceived leader status ratings for young male leaders compared to young female leaders. This provides suggestive evidence for the *subordinate male target hypothesis* (i.e., people with a preference for group-based hierarchy perceive especially male members of non-dominant groups as a threat to their dominant position; e.g., Sidanius & Pratto, 1999). Simple slopes are presented in Figure S5 (Aiken & West, 1991; McCabe et al., 2018).

Table S11. Results of Regression Analysis for Perceived Leader Status of Young Men in Study 2

		95% CI				95% CI		
	β	LL	UL	*p*	*β*	LL	UL	*p*
Main Effects (Step 1)								
Young Men vs. Young	-.09	-.24	.03	.13	-.10	-.25	.02	.10
**Young Men vs. Men (*H2*)**	**-.21**	**-.38**	**-.11**	**<.001**	-.21	-.38	-.11	<.001
Young Men vs. Young Women	-.12	-.27	.00	.05	-.11	-.26	.00	.06
Young Men vs. Women	-.29	-.48	-.21	<.001	-.30	-.48	-.21	<.001
(Young vs. Men) [a]	-.15	-.32	.01	.04	-.16	-.33	-.01	.03
SDO (centered)	-.08	-.21	.01	.08	-.22	-.52	-.02	.04
Two-way Interactions (Step 2)								
Young Men vs. Young x SDO					.03	-.12	.19	.62
**Young Men vs. Men x SDO (*H4*)**					**-.06**	**-.22**	**.07**	**.31**
Young Men vs. Young Women x SDO					-.15	-.34	-.02	.03
Young Men vs. Women x SDO					-.13	-.30	.00	.06
(Young vs. Men x SDO)[a]					-.16	-.38	.04	.10
R2	.07				.09			
F	6.61				5.04			

Note. N = 441. Standardized coefficients (*βs*) are reported. CI = confidence interval; LL = lower limit; UL = upper limit; SDO = social dominance orientation. Hypotheses tests are reported in bold.
[a] We only report the contrast between the young targets and men unique to the additional analysis.

Figure S5. Simple Slopes of Perceived Leader Status by Social Dominance Orientation Separated for Young Male Leaders and Young Female Leaders in Study 2

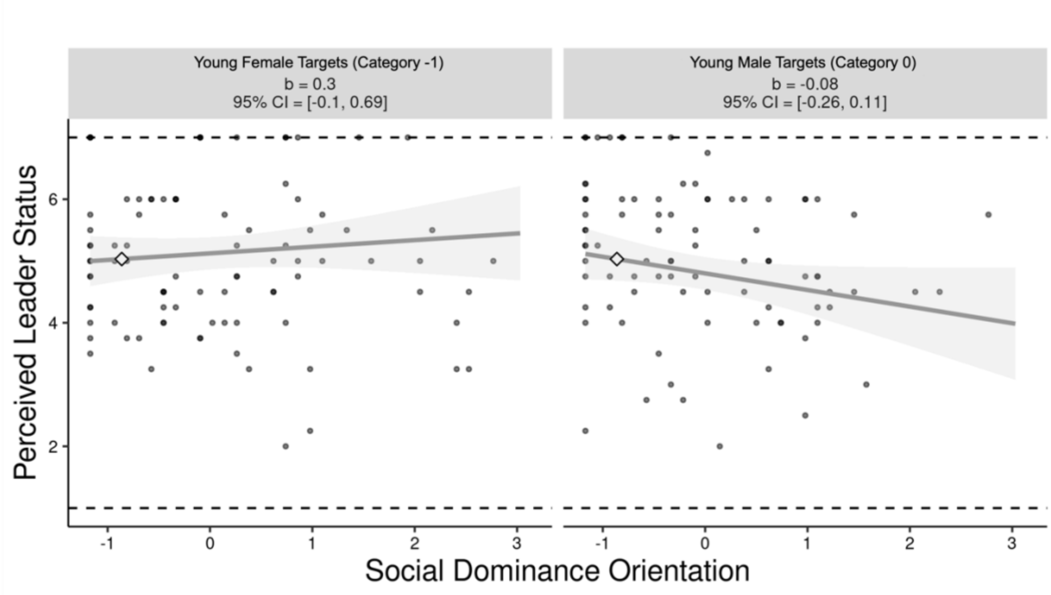

Note. The figure shows the computed 95% confidence region (shaded area), the observed data (gray circles), the maximum and minimum values of the outcome (dashed horizontal lines), and the crossover point (diamond).

We further explored the interaction effect of social dominance orientation and age groups on perceived leader status. Evaluators who exhibit a preference for group-based hierarchy and inequality (i.e., those with higher social dominance orientation; Ho et al., 2015; Pratto et al., 1994) are expected to assess young leaders more negatively than middle-aged or older leaders. To investigate this assumption, we considered social dominance orientation as a potential contingency factor in the leadership perception of young leaders compared to middle-aged and older leaders. We explored this interaction by running a regression analysis similar to our analysis of Hypothesis *H4*, presented in Table S11.

Young individuals were perceived as having less leader status than middle-aged and older leaders. Moreover, social dominance orientation moderated this negative relationship for young leaders compared to middle-aged leaders on perceived leader status. Simple slopes of social dominance orientation on perceived leader status, separated for young and middle-aged targets, are presented in Figure S6 (Aiken & West, 1991; McCabe et al., 2018). These simple slope tests reveal that social dominance orientation was significantly negatively related to perceived leader status for young targets, while it was non-significant for middle-aged targets.

Table S12. Results of Regression Analysis for Perceived Leader Status of Young Leaders in Study 2

	95% CI				95% CI			
	β	LL	UL	*p*	*β*	LL	UL	*p*
Main effects (Step 1)								
Young vs. Middle-aged Leaders	-.18	-.70	-.11	.01	-.19	-.73	-.14	.00
Young vs. Older Leaders	-.31	-.98	-.39	<.001	-.30	-.97	-.38	<.001
SDO (centered)	-.18	-.31	-.07	.00	-.34	-.56	-.15	<.001
Two-way Interactions (Step 2)								
Young vs. Middle-aged Leaders x SDO					-.20	-.64	-.06	.02
Young vs. Older Leaders x SDO					-.09	-.45	-.14	.29
R2	.10				.12			
F	10.23				7.33			

Note. N = 269. Standardized coefficients (*βs*) are reported. CI = confidence interval; LL = lower limit; UL = upper limit; SDO = social dominance orientation. The young leader condition was the condition of interest (i.e., young targets score a 0 on both dummies) that was compared with the middle-aged leaders (i.e., middle-aged targets coded -1) and older leaders (i.e., older targets coded -1).

Figure S6. Simple Slopes of Perceived Leader Status by Social Dominance Orientation Separated for Young Leaders and Middle-aged Leaders in Study 2

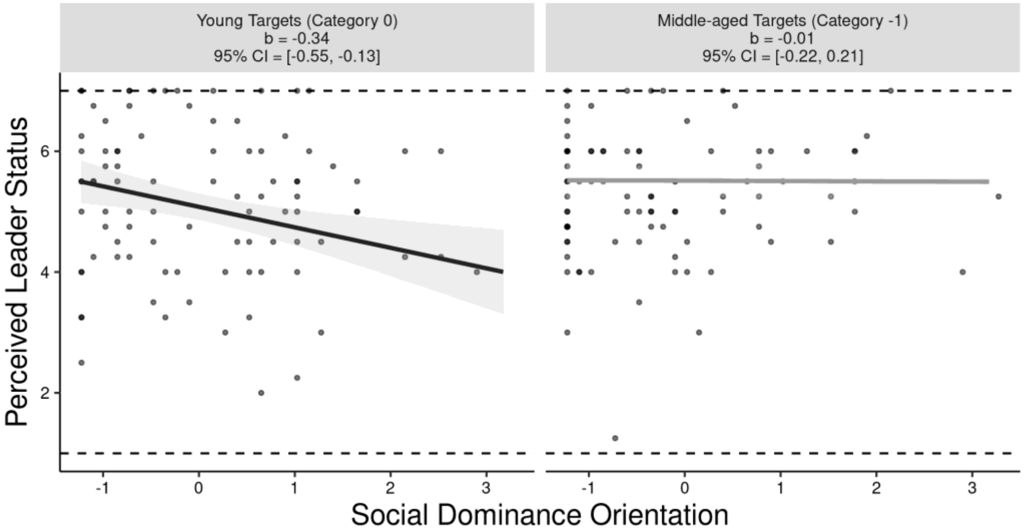

Note. The figure shows the computed 95% confidence region (shaded area), the observed data (gray circles), the maximum and minimum values of the outcome (dashed horizontal lines), and the crossover point (diamond).

5 References

Abele, A. E., Ellemers, N., Fiske, S. T., Koch, A., & Yzerbyt, V. (2021). Navigating the social world: Toward an integrated framework for evaluating self, individuals, and groups. *Psychological Review*, *128*(2), 290–314. https://doi.org/10.1037/rev0000262

Abele, A. E., Hauke, N., Peters, K., Louvet, E., Szymkow, A., & Duan, Y. (2016). Facets of the fundamental content dimensions: Agency with competence and assertiveness—Communion with warmth and morality. *Frontiers in Psychology*, *7*, #1810. https://doi.org/10.3389/fpsyg.2016.01810

Aichholzer, J., & Zeglovits, E. (2015). Balancierte Kurzskala autoritärer Einstellungen (B-RWA-6). *Zusammenstellung sozialwissenschaftlicher Items und Skalen (ZIS)*. https://doi.org/10.6102/ZIS239

Aiken, L. S., & West, S. G. (1991). *Multiple regression: Testing and interpreting interactions*. Newbury Park, CA: Sage.

Bailey, A. H., LaFrance, M., & Dovidio, J. F. (2019). Is man the measure of all things? A social cognitive account of androcentrism. *Personality and Social Psychology Review*, *23*(4), 307–331. https://doi.org/10.1177/1088868318782848

Giessner, S. R., & van Knippenberg, D. (2008). “License to fail”: Goal definition, leader group prototypicality, and perceptions of leadership effectiveness after leader failure. *Organizational Behavior and Human Decision Processes*, *105*, 14–35. https://doi.org/10.1016/j.obhdp.2007.04.002

Gündemir, S., Carton, A. M., & Homan, A. C. (2019). The impact of organizational performance on the emergence of Asian American leaders. *Journal of Applied Psychology*, *104*, 107–122. https://doi.org/10.1037/apl0000347

Hall, E. V., Hall, A. V., Galinsky, A. D., & Phillips, K. W. (2019). MOSAIC: A model of stereotyping through associated and intersectional categories. *Academy of Management Review*, *44*(3), Article 3. https://doi.org/10.5465/amr.2017.0109

Hayes, A. F. (2013). *Introduction to mediation, moderation, and conditional process analysis: A regression-based approach*. The Guilford Press.

Ho, A. K., Sidanius, J., Kteily, N., Sheehy-Skeffington, J., Pratto, F., Henkel, K. E., Foels, R., & Stewart, A. L. (2015). The nature of social dominance orientation: Theorizing and measuring preferences for intergroup inequality using the new SDO_7 scale. *Journal of Personality and Social Psychology*, *109*(6), 1003–1028. https://doi.org/10.1037/pspi0000033

Levin, S., Matthews, M., Guimond, S., Sidanius, J., Pratto, F., Kteily, N., Pitpitan, E. V., & Dover, T. (2012). Assimilation, multiculturalism, and colorblindness: Mediated and moderated

relationships between social dominance orientation and prejudice. *Journal of Experimental Social Psychology*, *48*(1), 207–212. https://doi.org/10.1016/j.jesp.2011.06.019

Lianidou, T., & Zheng, W. (2022). Leader diffuse status and leadership outcomes: Towards an integrative framework. *International Journal of Management Reviews*. https://doi.org/10.1111/ijmr.12316

McCabe, C. J., Kim, D. S., & King, K. M. (2018). Improving present practices in the visual display of interactions. *Advances in Methods and Practices in Psychological Science*, *1*(2), 147–165. https://doi.org/10.1177/2515245917746792

Offermann, L. R., & Coats, M. R. (2018). Implicit theories of leadership: Stability and change over two decades. *The Leadership Quarterly*, *29*(4), 513–522. https://doi.org/10.1016/j.leaqua.2017.12.003

Pratto, F., Sidanius, J., Stallworth, L. M., & Malle, B. F. (1994). Social dominance orientation: A personality variable predicting social and political attitudes. *Journal of Personality and Social Psychology*, *67*(4), 741–763. https://doi.org/10.1037/0022-3514.67.4.741

Preacher, K. J., & Hayes, A. F. (2004). SPSS and SAS procedures for estimating indirect effects in simple mediation models. *Behavior Research Methods, Instruments, & Computers*, *36*, 717–731. https://doi.org/10.3758/BF03206553

Prentice, D. A., & Carranza, E. (2002). What women and men should be, shouldn't be, are allowed to be, and don't have to be: The contents of prescriptive gender stereotypes. *Psychology of Women Quarterly*, *26*(4), 269–281. https://doi.org/10.1111/1471-6402.t01-1-00066

Rudman, L. A., Moss-Racusin, C. A., Phelan, J. E., & Nauts, S. (2012). Status incongruity and backlash effects: Defending the gender hierarchy motivates prejudice against female leaders. *Journal of Experimental Social Psychology*, *48*(1), 165–179. https://doi.org/10.1016/j.jesp.2011.10.008

Sibley, C. G., Wilson, M. S., & Duckitt, J. (2007). Antecedents of men's hostile and benevolent sexism: The dual roles of social dominance orientation and right-wing authoritarianism. *Personality and Social Psychology Bulletin*, *33*(2), 160–172. https://doi.org/10.1177/0146167206294745

Sidanius, J., & Pratto, F. (1999). *Social Dominance: An Intergroup Theory of Social Hierarchy and Oppression*. Cambridge University Press.
